# A Novel Hybrid Classification Model of Genetic Algorithms, Modified k-Nearest Neighbor and Developed Backpropagation Neural Network

**DOI:** 10.1371/journal.pone.0112987

**Published:** 2014-11-24

**Authors:** Nader Salari, Shamarina Shohaimi, Farid Najafi, Meenakshii Nallappan, Isthrinayagy Karishnarajah

**Affiliations:** 1 Department of Biology, Faculty of Science, University Putra Malaysia, Serdang, Selangor, Malaysia; 2 Department of Biostatistics and Epidemiology, School of Public Health, Kermanshah University of Medical Sciences, Kermanshah, Iran; 3 Department of Mathematics, Faculty of Science, University Putra Malaysia, Serdang, Selangor, Malaysia; Universitat Rovira i Virgili, Spain

## Abstract

Among numerous artificial intelligence approaches, k-Nearest Neighbor algorithms, genetic algorithms, and artificial neural networks are considered as the most common and effective methods in classification problems in numerous studies. In the present study, the results of the implementation of a novel hybrid feature selection-classification model using the above mentioned methods are presented. The purpose is benefitting from the synergies obtained from combining these technologies for the development of classification models. Such a combination creates an opportunity to invest in the strength of each algorithm, and is an approach to make up for their deficiencies. To develop proposed model, with the aim of obtaining the best array of features, first, feature ranking techniques such as the Fisher's discriminant ratio and class separability criteria were used to prioritize features. Second, the obtained results that included arrays of the top-ranked features were used as the initial population of a genetic algorithm to produce optimum arrays of features. Third, using a modified k-Nearest Neighbor method as well as an improved method of backpropagation neural networks, the classification process was advanced based on optimum arrays of the features selected by genetic algorithms. The performance of the proposed model was compared with thirteen well-known classification models based on seven datasets. Furthermore, the statistical analysis was performed using the Friedman test followed by post-hoc tests. The experimental findings indicated that the novel proposed hybrid model resulted in significantly better classification performance compared with all 13 classification methods. Finally, the performance results of the proposed model was benchmarked against the best ones reported as the state-of-the-art classifiers in terms of classification accuracy for the same data sets. The substantial findings of the comprehensive comparative study revealed that performance of the proposed model in terms of classification accuracy is desirable, promising, and competitive to the existing state-of-the-art classification models.

## Introduction

In the last decade, the extensive effect of classification models on decision making in various scientific fields including medicine, has attracted a lot of attention. Classification in the realm of research is the designation of an individual or an item to a set of classes so that the decision making is made based on the characteristic of that individual or the item. Successful classification depends on the two major factors of “how to select the most informative features” and the “classifier method”, especially in the field of medical classification. The widespread in congruency of features in this field has made the selection of a subcategory of the best factors of features more significant, and has given it a more effective and valuable role in the promotion of the performance of the classification model. Using a set of training patterns, in which the correct classification is known subcategory of classified observations called the training set, the classifier function organizes the classification. Thus, it is expected that proper selection and classification of methods at each stage would lead to a classification model with successful performance.

Following the first classification rule presented in 1936 by Fisher in statistical classification literature [1], various classification models have been proposed. Among them, the simple and efficient method for the implementation and understanding of non-parameterized classification was the k-Nearest Neighbor (k-NN) which has been well-received. For instance, in order to improve the classification accuracy, Weinberger and Saul [2] presented a developed algorithm of k-NN. In their proposed model, they used Mahalanobis distance as the criterion for distance determination. A developed hierarchical model of k-NN was introduced by Kubota et al. [3]. The high capability and sensitivity of this model in the fine discrimination of classes is noteworthy. Zeng et al. [4] have proposed a modified classification algorithm of k-NN whose underlying algorithm is local average and class statistics. That is, in addition to local information from k-NN of new non-classified data, general information about neighbors in each class is analyzed separately.

Artificial neural network is an efficient approach that in recent years has been considered by researchers as one of the most useful and applicable constructs in artificial intelligence. This is due to its numerous advantages such as being non-parametric (no requirement for any primary assumption on data), self-adaptiveness, ability to be generalized, and having a high capacity in modeling non-linear patterns. This approach is a functional technology that provides the user the possibility to obtain the best linear combination of features in order to achieve his/her goals including the classification of complex models, estimation of non-linear functions and prediction [5].

In the medical field, Olmez and Dokur [6] have proposed the use of artificial neural networks algorithm to classify heart beats. In their proposed model, they first selected the best features using dynamic programming; then, using artificial neural networks, they successfully classified heart beats into seven categories. To classify heart beat data, Rajendra A et al. [7] employed artificial neural networks and Fuzzy equivalence classifier. Qiu et al. [8] presented a model for classification of cervical cancer risk, using artificial neural networks. The findings indicated sensitivity and specifity of 98% and 97%, respectively. Salari et al. [9] used artificial neural network methods for prediction of late onset heart failure. In 2013, Salari et al. [10] used an integrated medical model based on artificial intelligence approach. The proposed model, was put forwarded for medical data classification.

However, traditional methods which are based on single technology were gradually replaced by hybrid models. Hybrid models which are increasingly getting noticed by researchers are a relatively new approach which include innovative, creative, and appropriate combination of several models for achieving a final common goal with a performance far better than traditional models based on single technology. The main idea behind these models is to benefit from the synergies among technologies. This characteristic provides the opportunity to learn about the exclusive strengths of each technology and can be used as a means of compensating for the deficiencies, and overcome limitations of each technology [11], [12].

The review of medical literature indicates that research on the application of hybrid models in the field of artificial intelligence is growing. Chakraborty [13] proposed an integrated approach for cancer classification and simultaneous gene selection. He argues that, because only a small part of the large number of genes in this field is suitable for discriminating between different types of cancer, it will be better if these two processes take place simultaneously. The application of this model is choosing findings among suitable genes and simultaneously developing a model of possible nearest neighbor for cancer classification. Ostermark [14] proposed a classification hybrid model by employing genetic algorithms, Fuzzy logic, and artificial neural networks. Aci et al. [15] presented a hybrid model with a combination of genetic algorithms, Bayesian methods, and k-NN. Their goal is to eliminate the data that are barrier to learning to achieve successful results in classification. Khashei et al. [16] proposed a hybrid model combining artificial neural networks and multiple linear regression. This model has been proposed for classification purposes, and for achieving higher accuracy and a more generalized application than the traditional artificial neural network models.

In 2014, Seera and Lim [17] also put forward a hybrid intelligent system for medical data classification. The proposed system consisted hybrid of the Fuzzy Min–Max neural network, the classification and regression tree (CART), and the random forest model. They concluded that the domain users (i.e., medical practitioners) were able to comprehend the prediction given by the hybrid intelligent system; thus accepting its role as a useful and efficient medical decision support system. Again, in 2014, Shao et al. [18] addressed the classification heart disease issue by combining the multivariate adaptive regression splines (MARS), logistic regression, artificial neural network, and rough set (RS) techniques. In initial step, the proposed hybrid model reduced the set of explanatory variables by using logistic regression, MARS, and RS techniques. Subsequently, selected variables was employed as inputs for the artificial neural network method in the process of classifying heart disease patients. Experimental results have shown the effectiveness of the proposed hybrid model to classify heart disease.

Forghani and Yazdi [19] came up with a hybrid model called “robust support vector machine-trained fuzzy system”. The proposed hybrid classifier established with a combination of support vector machine and Fuzzy if–then rules. Experimental results have shown the use of proposed approach results in very fast training and testing convergence time with good overall classification accuracy rate. In effect, this model had 63% of classification accuracy based on the Cleveland multi-class data set. Zhang and Zhang [20] suggested a hybrid method employing Rotation Forest in conjunction with AdaBoost. This model achieved 55.62% and 74.69% classification accuracies for the Cleveland multi-class and Pima's data sets, respectively. A classification model entitled “Forest Optimization Algorithm” was proposed by Ghaemi and Feizi-Derakhshi [21]. It was established by incorporating a few trees into the forests to improve the predictive accuracy of classifiers. This classification model attained 58.14% and 71.11% accuracies for the Cleveland multi-class and Pima's data sets, respectively. Zhang et al. [22] came up with a novel k-NN-based algorithm, 3N-Q, for enhancing the performance accuracy of k-NN classifiers. The reported experiment results demonstrated that 3N-Q is efficient and accurate for performing classification tasks.

The review of literature indicates that models with diverse applications based on various combinations of k-NN, genetic algorithms and artificial neural netwoks have been proposed for classification purposes. However, no measure has been taken for linking these three methods in the literature of classification models. Therefore, it can be argued that such an action is a novel approach that adds to the body of literature in this field. The present study aims to present a new model to appropriately link the above mentioned three methods. It is expected that the synergy resulting from the combination of these elements improves classification performance, especially in various medical fields.

This model begins with features prioritization using classification techniques that facilitate learning such as Fisher's discriminant ratio, and class separability criteria. In fact, Fisher's discriminant ratio is the criteria for features ordering in terms of discrimination ratio of both classes relative to each other whereas class separability criteria is the criteria for features classification in terms of discrimination ratio of each class relative to all other classes. Then, using high and unique capabilities of genetic algorithm in optimization, optimized arrays were produced so that the results of features classification, including previously classified arrays, were utilized as initial population of the genetic algorithm. Afterwards, using the modified k-NN method in parallel with a Developed Back Propagation Neural Network (DBPNN) method, the classification process was carried out according to optimization arrays of selected features by genetic algorithms. Finally, a method of Fuzzy class membership was applied to integrate and finalize decision making from proposed classes.

The new proposed model was tested with six data sets taken from the University of California Irvine (UCI) machine-learning repository as well as a dataset in the real world called Acute Coronary Syndrome Event — in Kermanshah, Iran (ACSEKI). From these data sets, four were on heart diseases, two on breast cancer and one on diabetes. In addition, the performance of the proposed new hybrid model was compared to some of the well-known classification models.

The rest of this study is organized as follows: section two presents the materials and methods including a brief explanation of each applied approach in the hybrid model; the framework and building process of the proposed hybrid model is described in detail; the model performance assessment process is presented and a detailed plan for the statistical evaluation of the model is provided. The results of the performance evaluation of the proposed model are discussed in section three comparing to some of the well-known classification models (based on seven different data sets) as well as statistical evaluation results. Finally, section four includes the conclusion.

## Material and Methods

In this section, first the attributes of each dataset is explained. Second, a brief review of concepts and methods of Genetic Algorithms, fuzzy class memberships, BackPropagation Neural Networks(BPNNs), and k-NNs is presented. Finaly, the proposed model is thoroughly described.

### 2.1 Data sets technical information

To test the proposed hybrid model in this study, widespread and different standard data sets from the real world were used. Among these data sets, four were on heart disease, two on breast cancer and one on diabetes. These data sets, briefly discussed here, are similar in terms of number and type of features, number of classes, and number of missing values.

One of the data sets applied in heart field is ACSEKI. Using the Euro Heart Survey on ACS, designed by the European Society of Cardiology, we registered all admitted patients referred to the Imam Ali hospital, the main center for cardiovascular care in Kermanshah, Iran. While the first Euro Heart Survey of ACS was conducted in 25 countries (in 2000–2001), the second survey involved 32 European countries. For the purpose of this registry, all hospitalized patients diagnosed with ACS during 2010–2011 were included. According to the standard protocol of this registry, all patients with unstable angina as well as those suspected of acute myocardial infarction were included.

A total of 2068 patients were enrolled in the study. They were divided into four groups based on ACS causes including STEMI, NSTEMI, UA, and other. In the case report, a form was completed for each patient by the attending physician. A data collection officer reviewed and checked each form for missing data. Table 1 shows the distribution of ACS causes.

**Table 1:** Class sample distribution in the ACSEKI dataset.

Class Label	Class Name	Frequency	Percent
1	STEMI	316	15.28
2	NSTEMI	461	22.29
3	UA	1196	57.83
4	Other	95	04.59
	Total	2068	100.0

For each subject, 266 clinical factors were collected consisting of numerical and nominal features. Based on numerous interviews with cardiologists, and examining references in relevant medical literature, 26 seminal features for classification of ACS were selected. These factors along with their description and data types are shown in Table 2.

**Table 2:** Detailed description of the recorded clinical features in the ACSEKI database.

No.	Name	Description	measurement scale
1	dmSex	Female, male	Nominal
2	age	Age in years	Numeric
3	BMI	Body mass index	Numeric
4	phMI	History of prior myocardial infarction	Nominal
5	phPriorAP	History of prior angina pectoris	Nominal
6	phCHF	History of congestive heart failure	Nominal
7	phStroke	History of stroke	Nominal
8	phLungDis	History of chronic lung disease	Nominal
9	phPCI	Prior PCI	Nominal
10	phCABG	Prior CABG	Nominal
11	phSmoking	Smoking status	Nominal
12	phDiab	Diabetes mellitus	Nominal
13	phHT	History of hypertension	Nominal
14	phHyChol	History of hypercholesterolemia	Nominal
15	phFamHist	Family history of Coronary Artery Disease	Nominal
16	adPresSymp	Predominant presenting symptom	Nominal
17	adHR	Heart rate	Nominal
18	adSysBP	Systolic blood pressure	Numeric
19	adtroponMax	Max measure of Troponin in first four tests	Numeric
20	adSTTchange	ECG STT changes	Nominal
21	adCKMB	CKMB mass elevated	Nominal
22	adSChol	Total cholesterol value	Numeric
23	adSCreat	Serum creatinine value	Numeric
24	adGluc	Glucose value	Numeric
25	adHaem	Haemoglobin value	Numeric
26	adRhythm	ECG rhythm	Nominal
27	dcDisDiag	Discharge diagnosis(class)	Nominal

The other six data sets used in this study are taken from the UCI, each taken from a different source. The Cleveland dataset collected in Cleveland Clinic Foundation is designed to determine the presence or the absence of heart disease in individuals based on some of their features. This dataset consists of 75 predictive features in addition to the type of the disease diagnosis feature that must be anticipated for new cases. From these 75 features, based on the expert views of heart specialists, 13 features that were more important in the disease diagnosis were chosen. The list and explanations of these features are presented in Table 3. Similarly, disease diagnosis feature includes five different classes; the first class belongs to healthy individuals while the other four classes belong to people affected with heart disease, according to the disease severity. The sample distribution of these classes is shown in Table 4.

**Table 3:** Detailed description of the recorded clinical features in the Cleveland dataset.

No.	Name	Description	measurement scale
1	Sex	Sex	Nominal
2	Age	Age	Numeric
3	Cp	Chest pain type	Nominal
4	Trestbps	Resting blood pressure	Numeric
5	Chol	Serum cholestoral	Numeric
6	Fbs	Fasting blood sugar	Nominal
7	Restecg	Electrocardiographic results during rest	Nominal
8	Thalach	Maximum heart rate achieved	Numeric
9	Exang	Exercise induced angina	Nominal
10	Oldpeak	ST depression induced by exercise relative to the rest	Nominal
11	Slope	Slope of the peak exercise ST segment	Nominal
12	CA	Number of major vessels colored by flourosopy	Nominal
13	Thal	The heart status	Nominal
14	class	Healthy = 0, sick type = 1, 2, 3, and 4 (beasd on severity of heart disease)	Nominal

**Table 4:** Class sample distribution in the Cleveland dataset (multiple classes).

Class Numbers	Class Name	Frequency	Percent
1	Healthy	164	54.1
2	Sick level 1	55	18.1
3	Sick level 2	36	11.9
4	Sick level 3	35	11.6
5	Sick level 4	13	4.3
	Total	303	100.0

In order to balance the distribution of classes in the Cleveland multi-class dataset, four classes of individuals with heart disease with different severity were combined with disease diagnosis feature. The result was the creation of the Cleveland two-class dataset along with disease diagnosis feature including two healthy and sick classes. The sample distribution of new classes is presented in table 5.

**Table 5:** Class sample distribution in the Cleveland dataset (binary class).

Class Numbers	Class Name	Frequency	Percent
1	Healthy	164	54.1
2	Sick	139	45.9
	Total	303	100.0

The Hungarian dataset is the last dataset in the field of heart disease which was used in this study. These data sets, which were collected in the Hungarian Institute of Cardiology, Budapest, consist of 249 instances. All of the features have a similar structure to that of the Cleveland two-class dataset. The sample distribution of these classes is indicated in table 6.

**Table 6:** Class sample distribution in the Hungarian dataset (binary class).

Class Numbers	Class Name	Frequency	Percent
1	Healthy	188	64
2	Sick	106	36
	Total	294	100.0

Another dataset used in this study is the Wisconsin Breast Cancer (WBC) which is provided by the researchers of the Wisconsin university. Between 1989 and 1991, Doctor Wolberg collected a group of digital images taken from fine needle aspirates (FNA) of biopsies from the breast of patients diagnosed with breast cancer. Afterwards, nine features of the images processed were measured. These features describe the characteristics of the cell nucleus shown in the image. The list of these nine features along with the diagnosis feature are illustrated in Table 7. Furthermore, disease diagnosis feature is comprised of two malignant and benign classes whose sample distribution is presented in Table 8. It should be noted that this dataset consists of 699 instances and also has missing values.

**Table 7:** Description of the recorded clinical features computed from the digital images of FNA of the breast masses in the WBC dataset.

No.	Name	Value
1	Clump Thickness	1–10
2	Uniformity of Cell Size	1–10
3	Uniformity of Cell Shape	1–10
4	Marginal Adhesion	1–10
5	Single Epithelial Cell Size	1–10
6	Bare Nuclei	1–10
7	Bland Chromatin	1–10
8	Normal Nucleoli	1–10
9	Mitoses	1–10
10	Class	Benign = 1, Malignant = 2

**Table 8:** Class sample distribution in the WBC dataset (binary class).

Class Numbers	Class Name	Frequency	Percent
1	Malignant	241	34
2	Benign	458	66
	Total	699	100.0

Another dataset used in the present study is Wisconsin Diagnostic Breast Cancer (WDBC). This dataset was also collected in 1995 by Wolberg et al. to diagnose breast cancer disease. To start with, digital images of FNA of biopsies from the breast of patients with breast cancer were collected. Then, ten features were measured using image processing methods to analyze the size, shape, and tissue of each cell nuclei, whose description is indicated in Table 9. Calculation ofthe mean, the standard deviation, and the extreme (mean of three of the extremes values) for each of theses 10 features resulted in 30 predictive features. Based on the the final result of this process, the total dataset of WDBC include 569 instances, and 30 predictive features along with disease diagnosis feature. Also, the disease diagnosis feature included malignant and benign classes whose sample distribution is shown in Table 10. It should be pointed out that this dataset has no missing values.

**Table 9:** Description of the recorded clinical features computed from digital images of FNA of the breast masses in the WDBC dataset.

No.	Name	Description
1	radius	mean of distances from center to points on the perimeter
2	texture	standard deviation of gray-scale values
3	perimeter	the border around cell nucleus
4	area	the amount of space that covers cell nuclus
5	smoothness	local variation in radius lengths
6	compactness	(perimeter∧2/area – 1)
7	concavity	severity of concave portions of the contour
8	concave points	number of concave portions of the contour
9	symmetry	the quality of being symmetrical
10	fractal dimension	(“coastline approximation” – 1)

**Table 10:** Class sample distribution in the WDBC dataset (binary class).

Class Numbers	Class Name	Frequency	Percent
1	Malignant	212	37
2	Benign	357	63
	Total	569	100.0

The Pima Indians diabetes data set is last data sets taken from UCI machine-learning repository. In Pima database, all of patients are Pima-Indian women at least 21 years old and living near Phoenix, Arizona, USA. The total dataset of Pima include 768 instances, and 8 predictive features per instance along with disease diagnosis feature including two healthy and sick classes. The predictive features along with their explanations are listed in Table 11. Note that, the healthy class is labeled as“negative to diabetes”, whereas the sick class is labeled as “positive to diabetes” whose sample distribution is shown in Table 12.

**Table 11:** Description of the recorded clinical features computed from digital images of FNA of the breast masses in the WDBC dataset.

Class Numbers	Class Name	measurement scale
1	Number of times pregnant	Numeric
2	Plasma glucose concentration (mg/dc)	Numeric
3	Diastolic blood pressure (mm Hg)	Numeric
4	Triceps skin fold thickness (mm)	Numeric
5	2-hour serum insulin (mu U/ml)	Numeric
6	Body mass index (weight in kg/(height in m^2^)	Numeric
7	Diabetes pedigree function	Numeric
8	Age (years)	Numeric

**Table 12:** Class sample distribution in the Pima dataset (binary class).

Class Numbers	Class Name	Frequency	Percent
1	Healthy	500	65
2	Sick (diabetic)	268	35
	Total	768	100.0

### 2.2 Genetic algorithms

Genetic algorithm is a randon search method that was introduced by John Holland and his students in 1975, based on Darvin's theory of evolution [23]. In this method, according to the law of survival of the fittest,“, the process begins with an initial population (response set) and, based on the target function, our goal of presenting the problem and an indicator of the individuals' performance (responses) continues in a repeating fashion in order to find better responses. The selection of the target function depends on the nature of the problem. In this study, the target function that follows the issue of more effective classification, is a classifier accuracy whose purpose is achieving maximum level. A simple algorithm for this approach is as follows:

- Step 1: Create the primary population
- Step 2: Evaluate the members of the primary population by fitness function
- Step 3: Select one or more pair of the population probabilistically based on fitness function
- Step 4: Crossover selected pairs from the third step
- Step 5: Randomly mutate some of the newly created members of the fourth step (within the permitted limit of the response set)
- Step 6: Evaluate all of the created population (new generation) based on the fitness function
- Step 7: End the operation in case the algorithm stopping criterion is met, otherwise repeat the operation from the third step

### 2.3 Backpropagation neural network

**Figure 1:**
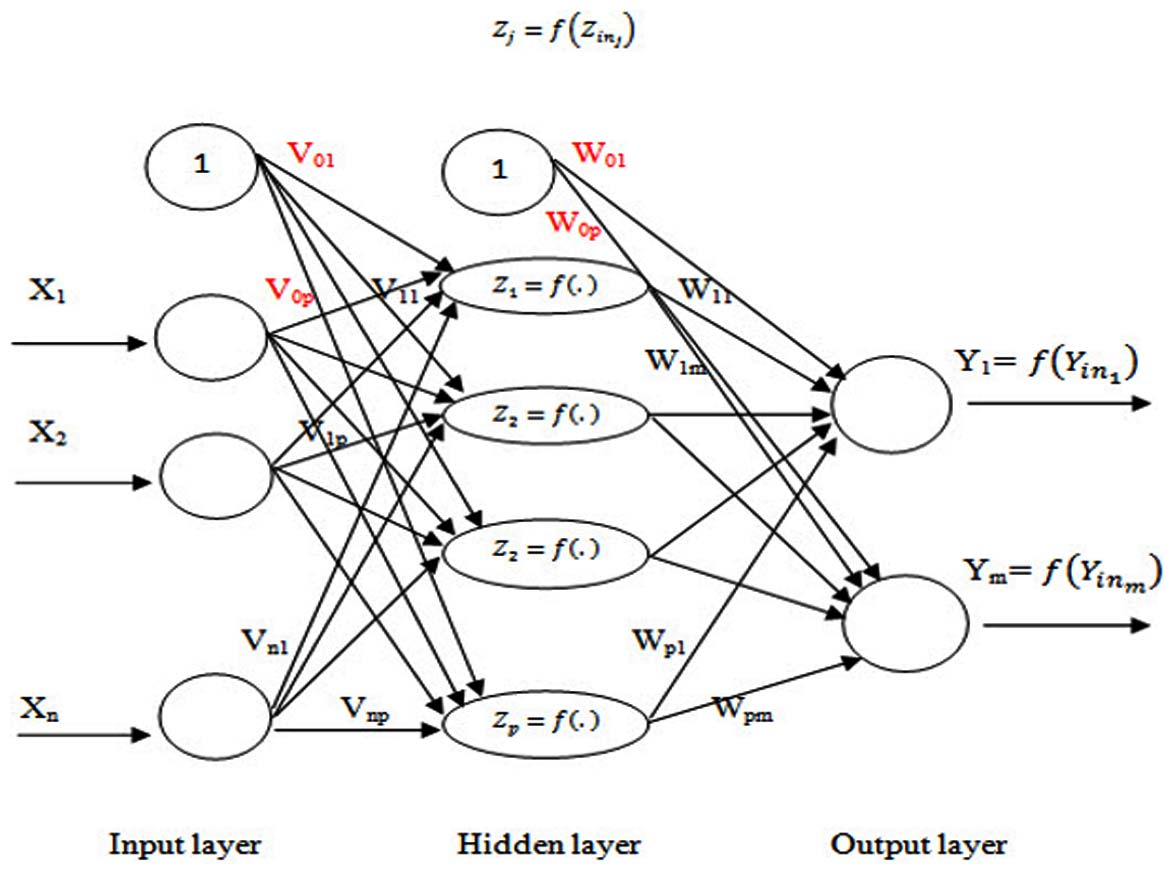
Backpropagation Neural Network.

One of the efficient methods in solving complicated problems is breaking them down to simpler subproblems that will be easier to comprehend and describe. It is these simple structures that describe the final complex system of a network when they are placed next to one another. Heb and Perceptron models are the simplest yet most efficient proposed arrangements for artificial neural networks consisting of an input layer, zero or a few hidden multilayers and an output layer. In this construct, all the neurons of a layer are linked to all the neurons of the next layer. This arrangement constitutes a network with complete connections. Nonetheless, due to the weakness of these networks in learning complex issues, BPNNs (i.e. a multilayer, feedforward network trained by backpropagation)were proposed by Werbos [24], Rumelhart et al. [25]. These networks create a good balance between memory power and generalization power. The general layout of this network is illustrated in Figure 1. Teaching this network includes three stages. First, calculation of the output corresponding to input (i.e. feed-forward phase). Second, error calculation and propagating to previous layers (i.e. backpropagation phase), and finally, adjustment of network weights(i.e. adjustment phase). The mathematical basis of backpropagation algorithm is an optimization technique called gradient descent. The gradient of a function is a direction in which the function ascends more rapidly, and consequently negative gradient is a direction in which the function quickly descends.

### 2.4 Fuzzy class memberships

Fuzzy sets theory was first introduced by Lotfizadeh in 1965 as the generalization of classical set theory. Following this, he presented a logic by the same name in the domain of new calculus [26]. The feature of lack of definite appears in various forms in all fields and phenomena. On the other hand, there are many inexact concepts around us that we refer to daily in various forms for which no precise quantity for their measurement could be found. Based on deductive thinking and considering various factors, the human brain in effect defines and evaluates statements in a way that their modeling to mathematical language and formulas remains a complicated task. Fuzzy logic is a relatively new approach proposed with the intent to overcome these increasing complexities as well as to design and model systems that require complex and developed mathematics using language variables and expert knowledge. It is an approach to solve problems that are far closer to human methods of thinking and learning. In keeping with this approach, many studies are carried out on pattern recognition and decision analysis [27]–[30].

In the realm of classification too, final decisions can be made based on this approach. Thus, in the present study, the final decision in determining the class of new data is made according to this approach and by presenting a probable array called class membership probabilities, which determines the allocation degree of this data to any specific class. Next, by integrating the created class membership arrays, we will reach a final decision making with fuzzy strategy. The details of administering this approach will be explained in details in the proposed new model section [31].

### 2.5 k-Nearest Neighbor Algorithm (k-NN)

One of the very simple machine-learning algorithms, in terms of classification implementation and understanding, is k-NN. Owing to good results albeit with a simplicity to solve many classification problems, this non-parametric method is still extremely popular in many research fields [32]. No training is given in this method and all of the training data are memorized. Therefore, it is known as an instance-based and lazy method [33]. To classify a new instance in this method, first the similarity, that is the distance between this instance and all other instances in the training set, is determined. Then, a k-NN in the training set closest to this new instance is chosen. Among this selected k-instance, the class with the most absolute frequency is considered as the new instance class. One can observe three key elements in this algorithm: a set of classified instances as training set, a criterion for calculating distance or similarity between instances (usually the Euclidean distance scale), and k [3], [34].

The performance of the k-NN algorithm can be improved by allocating a weight to each of k-NNs. This weight is chosen based on the distance of these instances to the new (observed) instance that should be classified, and usually has a reverse relationship with this distance. By choosing the weight, it will be possible to use all instances for classification instead of k-NN. This method is called distance-weighted k-NN algorithm and is effectively applied to different practical issues for inductive reasoning. This method is resistant to noise and is efficient when there is a lot of training data [33].

### 2.6 Proposed model

**Figure 2:**
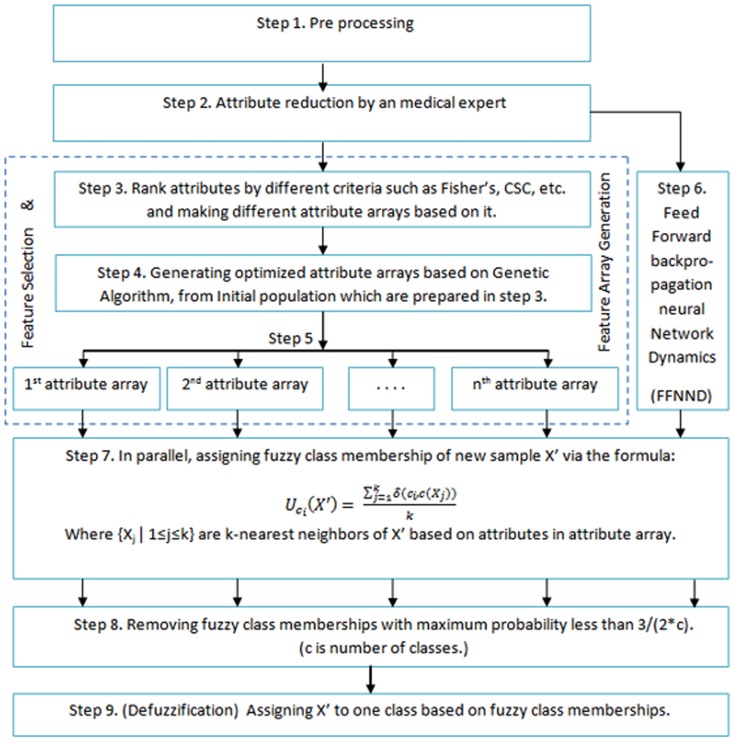
Implementation stages of the proposed new hybrid model.

The proposed novel hybrid model is created from the combination of artificial intelligence methods including genetic algorithms, DBPNN, modified k-NN, fuzzy class membership, and in conjunction with some pattern recognition methods to improve the classification accuracy. Implementation stages of the suggested model are presented in Figure 2. The full details of theses stages are subsequently discussed. Taking advantage of these benefits is one rationale behind using this algorithm as a part of proposed hybrid model in current study

- Step 1. Pre-processing: First, pre-processing is performed on all the data. That is, all quantitative features of dataset except the distinguishing variable of the disease class (response variable) are normalized. It should be pointed out that the dataset is considered as the n record of the m-dimensional, that is, it includes the m feature.
- Step 2. Medical feature selection: Based on expert views of skilled specialists, features that are important for disease diagnosis are selected.
- Step 3. Establishing prioritized feature arrays in different viewpoints:

The basic “majority voting” classification is considered as a controversial fundamental dilemma of the conventional k-NN algorithm [35]. The issue that when the class distribution is skewed can cause the samples of a more frequent class tend to dominate the prediction of the unclassified sample [36]. Essentially, this is because of the fact that they tend to be common among the k-NNs due to their large number. In this stage, in order to overcome this issue, the inherent bias of the majority class, are prioritized by different ways utilizing two pattern recognition methods, (i.e., the features defined in the previous step).

The rationale behind this strategy relies on the fact that in the present study, the nearest neighbors are found in different viewpoints (i.e., based on each of the generated prioritized feature arrays) which can greatly reduce the deficiency. It is because different combinations of features are resulted in different feature arrays —each with its own unique advantages. Ergo, for a new test instance vector, each feature array can lead to different nearest neighbors. Therefore, these feature arrays can be seen as different nearest neighbors' finder who are independent from each other. These independent arrays go to overwhelm the majority of voting difficulty.

The “Fisher discriminant ratio” [37], as a separability criterion, has derived from Fisher discriminant analysis. Due to its good capability in class separability viewpoint, it is a popular approach yet which is widely used in many pattern recognition problems; for example, see [38]–[40]. The Fisher discriminant ratio is a prioritized feature array according to the potential of each feature in discriminating between two specific classes. For instance, for a dataset with three classes c1, c2, and c3, features can be prioritized by three different ways. In the first method, features that discriminate c1 and c2 classes in the best way possible in a linear fashion receive higher priority. Accordingly, feature arrays are prioritized through two other methods for discriminating c1 and c3 as well as c2 and c3 classes. Generally, it can be argued that the Fisher discriminant ratio method builds 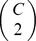 the arranged arrays of the features for a c class dataset.

Another method used in this study for features prioritization is the other “Class Separability Criteria” method. In this method, features' arrays are prioritized/ranked according to the potentiality of each feature in separating a specific class from other classes. This method, therefore, creates ranked arrays of features for each of the classes. The Class Separability Criteria can prioritize features using five different criteria, namely; t-test, relative entropy, Chernoff bound, Mann-Whitney test and receiver operating characteristic (ROC) curve (i.e., these criteria quantify the relationship between each band and the desired output).

Assuming a dataset with four distinct classes, using the Fisher discriminant ratio, six arrays will be built. For each, five criteria of class separability criteria with four arrays will be created, that is 6+(5×4) = 26. At the end of this step, feature reduction is performed, i.e. about one third of the elements of each array that have the lowest separability potential are eliminated.

- Step 4. Generating optimized features' arrays by genetic algorithm:

Based on the features' arrays obtained in the third step, we have attempted to create new generations of arrays that possess higher ability in classification tasks. The GAs was adopted to accomplish this goal. Since this algorithm was explained in section 2.2, its implementation is discussed here. It needs to be mentioned that choosing a good initial population is as a challenge in GA [41]. Therefore, using the ranked and the reduced feature arrays by different methods (i.e., Fisher discriminant ratio and Class Separability Criteria) can make the tolerable initial population for GA. Accordingly, the features' arrays produced in step 3 are considered as the initial population of this algorithm. Then, members of the initial population are evaluated by the fitness function (or the target function). Since our goal in the next stage is the application of the results of this genetic algorithm in a k-NN classification so as to increase its accuracy, therefore the best fitness function of this genetic algorithm that evaluates the members of population can be the k-NN classifier. Thus, the k-NN method performs the classification based on feature arrays. The efficiency of the k-NN approach for each of the arrays determines the array's fitness.

Next, weighted random selection is performed on the members of the population using the Roulette wheel approach. The value of these weights is determined based on the results obtained from the fitness function. Now, crossover operator is performed on each pair of the feature array that was selected as parents to produce the children of the new generation. In this study, the one-point crossover method was used from among various crossover methods. That is in each pair of features' array selected as parents from a randomly selected point, part of the two arrays are exchanged with each other. If there is a specific feature in two arrays of features selected for crossover, it is possible that, due to crossover, the children of the new generation contain repetitive features. The mutation operator (that randomly replaces a specific feature with a value within its permissible limit) is used as a solution for solving this problem. By performing these steps, the first repetition of algorithm is finished, and a new generation with combination of children and members of the initial population, which had a higher fitness, is created. By utilization this generation as the next repetitive primary population, the algorithmic steps continues until reaching the stop condition. At the end of the genetic algorithm steps, the process of reducing and selecting the features does, in effect, finish, and the obtained results are the best feature arrays that could assist the classification in the next step.

- Step 5. Classifying new instance vector 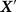 using modification k-NN

The classification is implemented using the k-NN method with a modification in its algorithm, that is in the final decision making step of determining class label of the new instance. For classification of every new instance vector 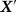, similar to conventional k-NN method, first it calculates the distance (usually Euclidean) of this instance to all the other instances of the training set (whose class label is known). Second, these calculated distances are sorted. Then, k of the closest instances/neighbors of the training set to the new instance is selected. In conventional k-NN, the final decision about determining the class label of the new instance vector is made based on the majority vote of these k closest neighbors. While in the present study, this step of the k-NN improved by the allocation of Fuzzy class membership to the new instance/input vector (i.e., incorporating Fuzzy set theory (Fuzzy Logic) instead of crisp set theory (Boolean Logic) in k-NN). That is, an array with a dimension equal to the number of classes is built (i.e., “Fuzzy class membership array”), the votes of every of classes is inserted into the array (without exertion of the majority of votes). In effect, the Fuzzy class membership arrays is calculated through equation 1:
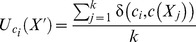
(1)where k is the number of nearest neighbors and Xj, is j^th^ the nearest neighbor of 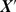 in the k-NN method and 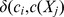 is the indicator function. Hence, this array is indicative of the degree of belongingness of the new instance 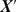 to each class (i.e., the results of dividing the number of neighbors belonging to each of the classes by the number of k). In this stage, the class membership array is calculated for each of the output feature's arrays of GA. Subsequently (in steps 8 and 9), these arrays along with the Fuzzy class membership array derived from DBPNN are integrated together to predict final class label of this new instance 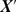.

- Step 6. Introducing Dynamic BackPropagation Neural Network

The main aim of this phase is the introduction of a newly improved neural network that advances the classification process in parallel with the k-NN method that was improved in the previous step. It is expected that, with regard to the different construct of these two classification approaches, the classification be improved using the high potential resulting from the synergy between the elements of these two classification approaches. In effect, the presented model is a dynamic neural network that in this study is called the DBPNN. The difference of the new method with the traditional BPNN method is that in each epoch, the transfer function is made dynamic in a way that the learning speed and accuracy is increased remarkably. Usually, functions such as log-Sigmoid (logistic) and/or tan-Sigmoid are the most common transfer functions used in BPNNs. These functions, owing to their desirable characteristics, have shown an appropriate performance in feedback neural networks. One of their benefits is that derivative of these functions is obtained according to the function, that is (Equations 2–3): 
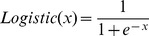
(2)

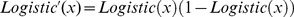
(3)


**Figure 3:**
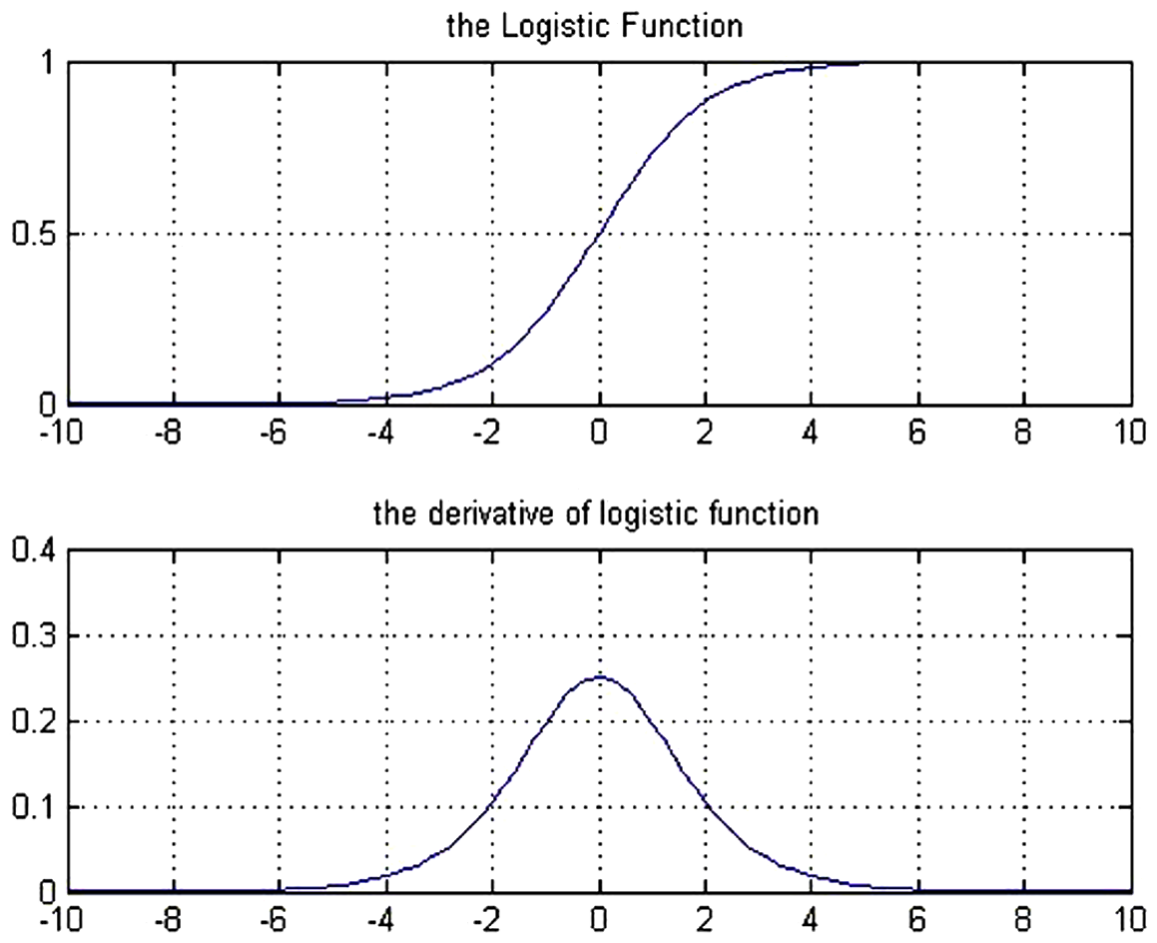
Graph of the Logistic function and its derivative function.

The graph and the derivative of this function are shown in Figure 3.

To increase the speed and the learning accuracy, the active domain is made dynamic in this study. In logistic function, the domain is (−∞ ∞) and the range is [0 1], but its active domain is limited to the range [−4, 4]. In other words, this function takes the value around zero for the values of range (−∞ −4), and a number around 1 for the values of range (4 ∞) (with a maximum level of error of 0.018; that is the output equals to 0 or 1, ignoring this error).

In the new method, DBPNN, there is an attempt to modify sigmoid function parameters in each epoch in a way that the active domain correspond to network inputs and weights. Therefore, we try to achieve this goal, step by step, by the application of suitable modifications. As mentioned above, the range of the Sigmoid logarithm function is the interval [0 1]. Now, we intend to define a function with Sigmoid logarithm nucleus whose range interval is [−1 1]. To this end, we must define a map from [0 1] to [−1 1]. Assuming that this map is a linear modifier, we can consider 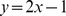, and consequently we have equations 4–6. 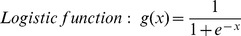
(4)where; 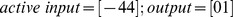


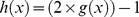
(5)

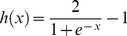
(6)where; 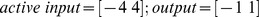


As mentioned previously, the Sigmoid logarithm active domain is in the range [−4 4]. By making an appropriate change, we now intend to define another function that could change its active domain to any [m M] desired range. In other words, to change every [m M] desired range to range [−4 4] (Equations 7–8). 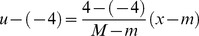
(7)

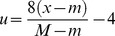
(8)


However, by comparing 6 and 8, we will have equations 9–13.



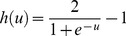
(9)

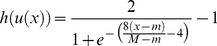
(10)

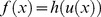
(11)

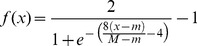
(12)

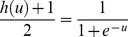
(13)


The derivative of function f (.) is presented in equations 14–18.
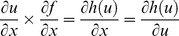
(14)

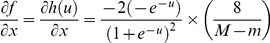
(15)

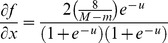
(16)

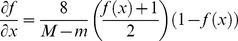
(17)

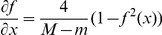
(18)


As can be seen, a function with Sigmoid logarithm nucleus is proposed whose active domain can be dynamically changed to any desired range [m M]. In addition, the derivative of this function can be presented as a function of Sigmoid logarithm. The next goal is to obtain a function that could dynamically change the output range to any desired range of [a b]. To achieve this goal, that is transferring the range of the function from [−1 1] to [a b], equations 19–22 are presented.
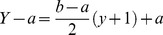
(19)

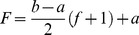
(20)

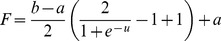
(21)

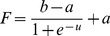
(22)


Now according to the presented introduction, we will attempt to organize the above-mentioned materials to propose a function with nucleus of Sigmoid logarithm function so that the active range accept any desired range of [m M] instead of the range [−4 4], and the desired output be any desired range of [a b] instead of the range [0 1], that is:
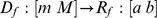



Assume that we have equations 23–25:
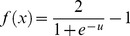
(23)

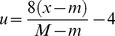
(24)

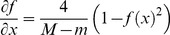
(25)


The transferred function is (Equations 26–28):
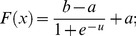
(26)

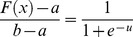
(27)

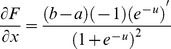
(28)


The first derivative of this function is obtained through (Equations 29–31):
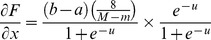
(29)

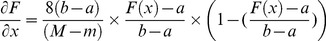
(30)

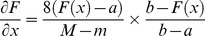
(31)


Therefore, along with its derivative, the final function with the Sigmoid logarithm nucleus function (in effect, Sigmoid logarithm function with new parameters) for transferring the range [m M] to the range [a b] is shown in equations 32 and 33. 
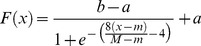
(32)

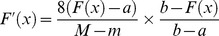
(33)


**Figure 4:**
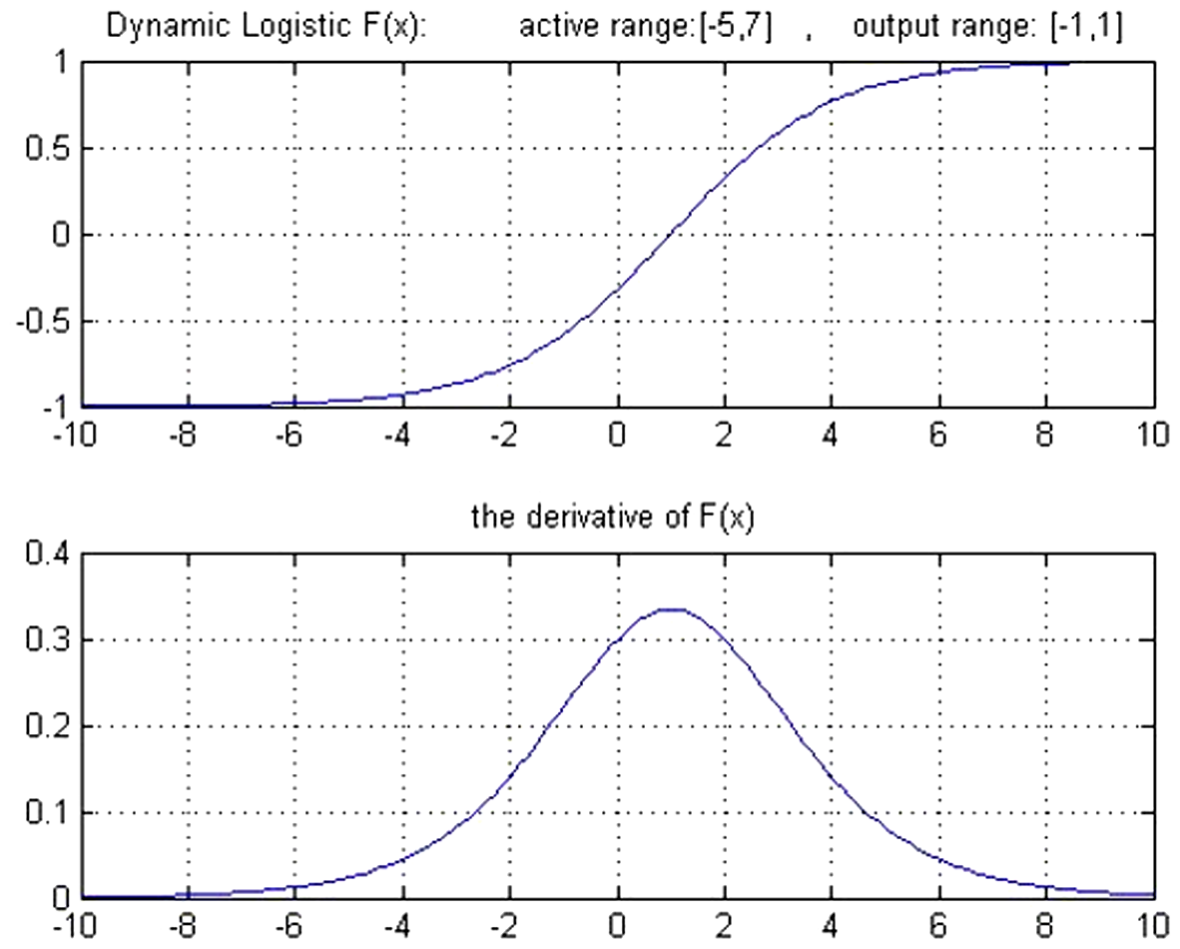
Graph of the dynamic logistic function and its derivative function for active input range [−5 7] and output range [−1 1].

**Figure 5:**
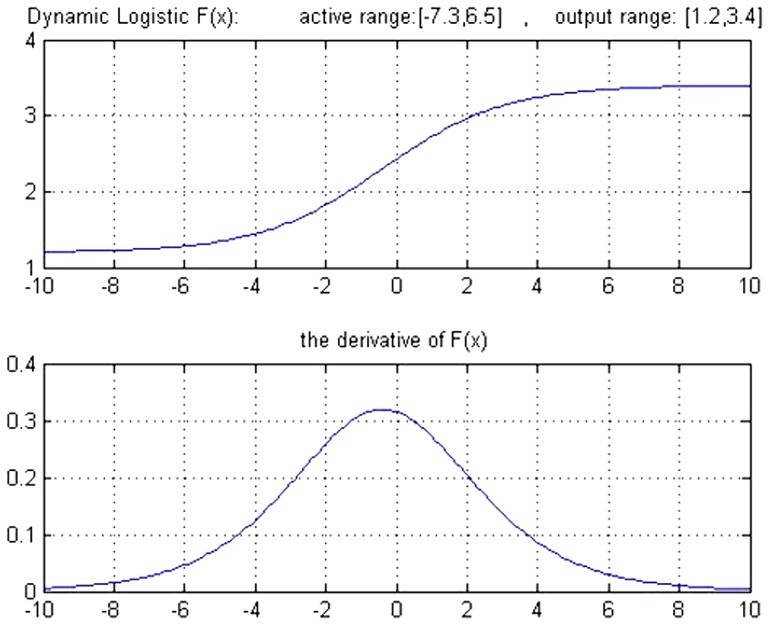
Graph of the dynamic logistic function and its derivative function for active input range [−7.3, 6.5] and output range [1.2, 3.4].

The shape of this dynamic logistic function and its derivative for different inputs and outputs are shown in Figures 4 and 5.

This final modification function has a dynamic capability that presents a function of the Sigmoid type by defining input and output ranges. Additionally, the derivative of this function is calculated according to that function at every point. Another benefit of this function is that its derivative is maximum in the center of its definition range which can be very important for neural networks. This is because, for values farthest from real output value, the network output considers the longest steps resulting in quicker network learning. It should be mentioned that in common networks, if the length of the step is large, the network cannot precisely regulate the weights and a large error will always exist. Conversely, if the length of the step is small, the possibility of entanglement in local minimum increases and also, network learning in this local minimum is very slow. Therefore, functions such as the Sigmoid logarithm or the Sigmoid tangent, whose derivative increases in case of high error, were introduced.

A criticism posed on static backpropagation networks was that regular transfer functions (such as tan-Sigmoid and/or log-Sigmoid) that these networks use, practically function well only in a limited range of their domain known as active range. Out of these ranges, the network has a rather fixed output and the derivative in these distances is also very close to zero.

The application of this dynamic function presented as transfer function of this network can be a giant step in removing this deficiency in a way that in each epoch, an appropriate active range [m = min M = max] is determined for each transfer function and changes it to the range [−1 1] where the range [m M] is determined according to the network weights. We have used this developed neural network on our own algorithm to implement the classification in parallel with the modified k-NN method. In this neural network, features that are important in the diagnosis of the disease based on the viewpoints of expert specialists (results of step 2) are considered as input, and the degree of belongingness of a new instance vector to every specific class as output.

- Step 7. Assigning Fuzzy class membership array to new instance vector 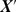 in DBPNN

Herein, at first, it should be mentioned that the proposed hybrid classifier has been designed in a way that, like some classifiers, e.g., Naive Bayes, assigns instance probability or score to each new case, which indicates its degree of belongingness to each specific class (i.e., Fuzzy class membership). In other words, this classifier is like a scoring or a ranking classifier and not a discrete one. In fact, “Fuzzy class membership array” is an array with a dimension equal to the number of classes. This array is indicative of the degree of belongingness of the new instance vector 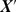 to each specific class. For instance, we assume that in a classification problem with five classes, the proposed classifier for a new case assigns the following belongingness degrees, which are shown in Table 13. As these belongingness degrees are independent, it is not necessary for their sums to be one.

**Table 13:** A typical Fuzzy class membership array.

Number of classes	1	2	3	4	5
**belongingness degrees**	%1.2	%42	%21	%56	%78

- Step 8. Removing Fuzzy class membership array with highest small belongingness degree

In this and next steps, “Fuzzy class memberships” tries to select one of the classes as the predicted class for the new instance vector 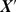. To this end, let us consider a classification problem with “c” classes problem, if the highest belongingness degree to classes in a class membership array is less than 3/(2×c), this array will not be used in decision making. This is because membership array of classes with highest small belongingness degree (i.e., less than 3/(2×c)) does not probably have a good decision making power, which could stem from the lack of appropriate selection of suitable neighbors.

- Step 9. Assigning a class label to new instance vector 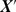

In this step, the difuzzification operation i.e., changing the output of Fuzzy viewpoint to a crisp form is carried out. Based on the integration of the results of the class membership arrays obtained from DBPNN and k-NN classifiers, the final decision process is reached in predicting the instance vector class label. In order to do the integrating process in the best possible way, a weight is applied to this process based on the number of class membership arrays created by k-NN (i.e., it has been adopted a weighted average). Various methods are proposed for the subject of integration in the Fuzzy theory. In this study, the approach taken to predict the class label of the new instance 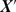, is based on a class that has the maximum total belongingness degree. Therefore, the degree of belongingness to any specific class is averaged up in all the class membership arrays.

### 2.7 Performance assessment

#### 2.7.1 Model validation

Model validation is possibly the most important step in the model building sequence. There are various resampling-based model validation methods with cross-validation being the most popular [42]. In the process of model construction, model selection, and model validation, cross-validation assesses a model based on error/accuracy-rate estimation (i.e. estimation of the generalization error/accuracy). In the current study, was used repeated random sub-sampling cross-validation method from among others cross-validation method (i.e. holdout, k-fold, and leave-one-out cross-validation method) [43], [44].

In this approach, the dataset is split into two sets of training and test. The training set is used to find the model's parameter of interest (i.e. model construction and selection). In addition, test sets are used to evaluate the generalizability performance of the final classifier/model. The process of train–test are repeated several times using different random samples which can be a common way to reduce any bias. Finally, the estimate of the overall error/accuracy rate is derived by averaging over all the separate error/accuracy rate estimates produced from different iterations [45].

In pattern recognition problems, the potential benefits of cross-validation method can help prevent two fundamental problems. The problems are (i) overfitting of final model (i.e. final model is unable to generalize to unseen data) and (ii) the error rate estimate will be overly optimistic (i.e. lower than the true error rate). On the other hand, if we want to select the classification model and estimate the error rate/accuracy rate simultaneously, three-way data splits procedure should be applied during the cross validation process. That is, the data should be divided into three disjoint (non-overlapping) sets namely training, validation, and test sets.

In this approach, the training set is used for learning, i.e. to optimize the tuning parameters of the model/classifier (e.g. in MLP, in order to determine the optimal weights and the bias with the back-propagation rule). The validation set is used to optimize the structural/regularization parameters of the model/classifier (e.g. in MLP, in order to determine the optimal number of hidden units and a stopping point of the algorithm). The test set is used only to estimate the error/accuracy rate (assessing the performance) of the final model. In other words, once both tuning and regularization parameters of the model/classifier have been optimized, the testing process will start. The procedure, using a three-way data split method is presented as follow Dougherty [42]:

- Step i: Divide the available data set into training, validation, and test sets.
- Step ii: Choose the architecture and training parameters.
- Step iii: Train the model using the training set.
- Step iv: Assess the model using the validation set.
- Step v: Repeat steps ii–iv using different architectures and training parameters.
- Step vi: Select the best model as the final model i.e. based on the estimate of the overall error/accuracy rate on validation set.
- Step vii: Evaluate the final model using the test set.

It should be noted that, this procedure is based on a holdout method. If other cross-validation method is utilized, steps iii and iv should be repeated for each of the k (i.e. k is number of folds in k-fold method or it is the number of times to repeat the random sub-sampling method).

#### 2.7.2 Performance evaluation criteria

Performance evaluation is one of the major introductory steps of a new classification model. The performance evaluation criteria are usually built from a confusion matrix, which can be categorized into two major approaches: numerical and graphical. Numerical approaches summarize and quantify the performance of a final classifier (i.e. fully-trained model) in a single number, whereas graphical approaches portray the performance in a plot. In this study, numerical approaches have been adopted as performance evaluation criteria. A confusion matrix or contingency table is a C×C matrix/table, in which each row is indicative of the actual class and each column of this matrix indicates the predicted class. Indeed, in a confusion matrix, the diagonal elements indicate how many subjects have been correctly classified, whereas the off-diagonal elements indicate how many subjects have been misclassified. Therefore, larger diagonal elements and smaller off-diagonal elements of the matrix would reflect a higher level of classification power and vice versa.

#### Binary-class' performance evaluation criteria

Various numerical-based criteria are used to quantify the performance of a binary-class classifier (i.e. measure of the generalization capability of classifiers) described in the literature. The conventional data layout for the 2×2 confusion matrix, used to calculate numerical-beasd criteria, are shown in Table 14. Here, TP and TN stand for the number of positive and negative examples, respectively, (i.e. sick and healthy people) that are classified truly while FP and FN stand for the number of positive and negative examples, respectively, that are classified falsely.

**Table 14:** The conventional data layout for the 2×2 confusion matrix.

		Predicted class		
		Positive	Negative	
Actual class	Positive	**TP**	**FN**	TPR
	Negative	**FP**	**TN**	TNR
		PPV	NPV	

**Figure 6:**
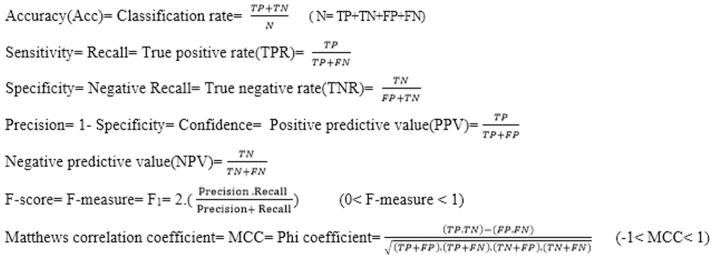
The definitions of confusion matrix-derived accuracy measures.

However, on the basis of the results of Table 14, it is possible to derive many of the classification performance metrics defined in Figure 6 [46].

Accuracy is the rate of correctly classified subjects and its appeal in presenting a single summary estimated to assess the overall effectiveness of a classifier which has been become the most commonly used measure for these purposes. However, in classification problem with im-balaced classes, accuracy is not a proper criterion [47]. Because, the overall accuracy does vary with the classes' frequency frequency changes (i.e. disease prevalence), it is obviously presented in equations 34–36.
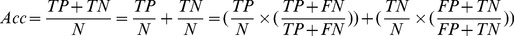
(34)

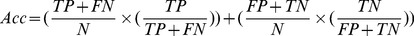
(35)

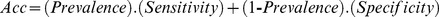
(36)


Where, Prevalence =  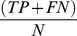 and (1-Prevalence) =  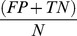


Accordingly, the classification accuracy is not sufficient as a performance evaluation of the classification models [48]. Sensitivity denotes the percentage of actual positive cases (i.e. sick subjects) correctly recognized by the classifier whereas specificity denotes the percentage of actual negative cases (i.e. healthy subjects) correctly recognized by the classifier. Indeed, both latter criteria, qualified the classifier' performance on different classes. Negative Predictive Value (NPV) is the part of predicted negatives that are the actual negatives. The precision (i.e. positive predictive value) is the part of predicted positives that are the actual positives. Precision and Recall are two criteria which are opposite to each other in terms of effectiveness: aa precision increases, recall usually decreases, and vice versa. The F-measure criterion, which takes precision and recall into account, is the harmonic-mean of these two [46]. The values of F-measure lie in the interval (0, 1) and larger F-measure values denote higher classification performance. The MCC is a correlation coefficient on the basis of the true and false positives and negatives, which can be used to as a performance evaluation criterion in binary-class classifications [49]. It returns a value between −1 and +1; where −1, 0 and +1 indicate the worst possible classification, a completely random classification and a perfect classification, respectively.

#### Multi-class' performance evaluation criteria

Researching in the context of performance evaluation criteria of multi-class classification is still an open topic because in the literature most of the criteria are originally designed only for binary-class tasks, although significant efforts have been carried out to develop them in the last few years [50]–[52]. The results of these efforts have introduced an expanded form of the criteria, which have been developed and adapted for evaluating multi-class classification in one of the following two strategies: one vs. one and one vs. rest. In one vs. one strategy, the performance is evaluated by measuring the capability to discriminate among the subjects of one class (i.e. considered as a positive class or a reference one), from subjects of another classes (i.e. considered as negative class). The second strategy is called one vs. rest (i.e. one vs. all), in which performance is assessed by measuring the capability to discriminate between subjects of one class (i.e. considered as positive class or reference class), from subjects of all the other classes (i.e. considered as negative class). The one vs. one and one vs. rest strategies produce a separate 2×2 confusion matrix for each “pair of classes” and for “each class”, respectively, with a corresponding set of values of classification performance criteria. Ultimately, the desired criteria can be achieved by combining the results appropriately. It should be noted that, in the present study the one vs. one strategy was used.

In this section, the definition of the developed form of some of the performance measures for multi-class classification problems are briefly addressed. As mentioned earlier, the F-measure is the harmonic mean of recall and precision, in multi-class problems, Pi, Ri and Fi are stand for precision, recall and F-measure for class (i.e. class reference) respectively, which is defined in Equations 37–39: 
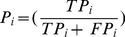
(37)

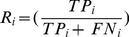
(38)

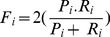
(39)


Here, TPi stands for the number of examples from class (i.e. reference class) “i” that are classified truly to class “i”, FNi stands for the number of examples from class “i” that are classified falsely to another class and FPi stands for the number of examples that are classified falsely to class “i”. Ultimately, the overall precision, recall, and F-measure of the multi-class classification problem can be obtained by two different kinds of average, namely, micro-average and macro-average. The computation of micro average precision (P-micro), micro average recall (R-micro) and micro average F-measure (F-micro) are done by Equations 40–42 respectively. In fact, F-micro represents a harmonic mean of P-micro and R-micro.
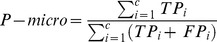
(40)

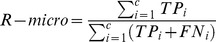
(41)

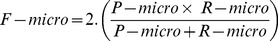
(42)


The computation of macro average precision (P-macro), macro average recall (R-macro) and macro average F-measure (F-macro) are done in two steps for each one. Firstly, computing precision, recall, and F-measure locally over each reference class (by equations 37–39 respectively); secondly, taking the average of all the obtained values (i.e. based on each reference class), for each criterion by Equations 43–45 respectively
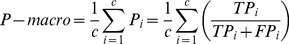
(43)

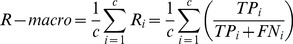
(44)

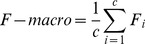
(45)
*where*; 
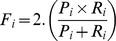
 (45-1)

The confusion entropy (CEN) is an entropy theory-based criterion, which has been recently introduced for evaluating the performance of multi-class classifiers. The evaluation criterion thoroughly takes the advantage of the misclassification information of the confusion matrix. In fact, it evaluates the confusion level of the class distribution of misclassified samples. In a c-class classification problem with confusion matrix c×c, the CEN is defined by Equations 46–47:
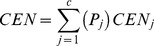
(46)

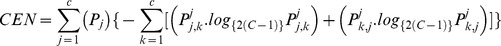
(47)


Where, 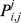
is defined as the misclassification probability of classifying the samples of class i to class j subject to class j and is calculated by Equation 48; 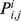 is defined as the misclassification probability of classifying the samples of class i to class j subject to class i calculated by Equation 49; and also 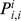 = 0; 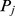 is defined as the confusion probability of class j calculated by Equation 50:
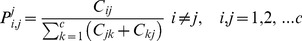
(48)

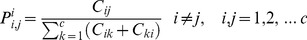
(49)

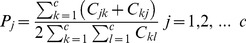
(50)


In binary-class classification, the CEN can be directly calculated by using confusion matrix results by Equation 51Jurman et al. [51]:
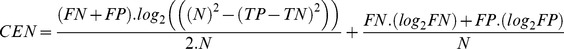
(51)where, N = TP+TN+FP+FN.

The CEN takes the value between 0 and 1; where 0 indicates a perfect classification whereas 1 indicates that the worst possible classification i.e. an interpretation in opposite dirction of other evaluation criteria. Thus, in order to solve this issue (i.e. to simplify and be perfectly comprehensible interpretation of the CEN result in comparison with other evaluation criteria), in this paper the measure for CEN is subtracted from 1. Jurman et al. [51] showed that CEN should not be reliably used in the binary-class classification. In the binary-class case, CEN can even take values greater than 1. Therefore, in the present study it has been refrained from employing the CEN as a performance evaluation criteria in the binary-class cases.

The MCC is another performance criterion, which has been developed and adapted to multi-class problems [51], [53] and it is calculated by Equation 52.
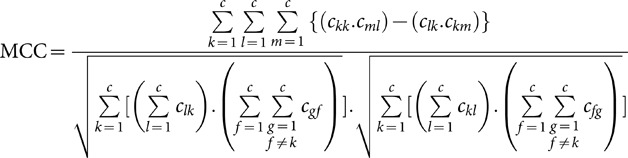
(52)


### 2.8 Statistical tests

One might rather say that the statistical analysis is an integral part of any scientific research [54]. Nevertheless, little attention has been paid to these valuable procedures in the artificial intelligence-based studies area [55], [56]. Thus, surprisingly, very few studies can be found recently in the literature that have been performed the statistical analysis. Therefore, in the present study, in order to improve the evaluation process of the performance of the novel hybrid model, statistical analysis is performed.

Statistical methods developed to carry out statistical analyses, from a methodological point of view, which can be categorized as parametric and nonparametric methods [54]. In fact, as a general rule, statistical inference procedures, which are used for evaluating a dependent variable measured by a nominal or ordinal scale, are categorized as nonparametric procedures, whereas those which are used to evaluate a dependent variable measured by an interval or ratio scale are categorized as parametric procedures. It should also be mentioned that there are other underlying assumptions, i.e. independence, normality, and homogeneity, which need to be checked for a more safe and prudent usage of parametric tests [55]. However, there is a trade-off here. Even though parametric tests are generally more powerful than their nonparametric analogs, many researchers believe that if one or more of the fundamental assumptions of a parametric test are violated, the power advantage of the parametric test may be negated, thereby the statistical analysis loses credibility [54], [57]. Accordingly, in such circumstances a prudent approach can be employed a suitable nonparametric tests.

#### 2.8.1 Preliminary analysis (Checking the conditions for a safe use of parametric tests)

In this section three underlying assumptions, which need to be checked for a more safe usage of parametric tests is briefly described. By definition, the two events are independent if the occurrence of one of them does not modify the probability of the occurrence of the other [58]. In our present case, it is obvious that the independence of the obtained results is derived from independent runs of the algorithm with randomly generated initial seeds. Ergo, it is necessary to check the rest of the fundamental assumptions of a parametric test.

A random variable X has the normal or Gauss distribution with mean µ and standard deviation σ (i.e., X ∼ N (µ, σ)) if its probability density function is given by Equation 53 [58]. 
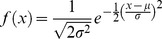
(53)where 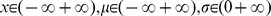


In the present study, in order to verify the normality hypothesis, the Shapiro-Wilk test was used as a preliminary analysis. This test is the most effective omnibus test that is able to find out departures from normality caused by either skewness or kurtosis or maybe both [59]. In fact, the Shapiro-Wilk test employed a weighted sum of ordered observations to quantify how the observations are far from a Normal distribution. Subsequently, the p-value drives through the sum of the squares of these disparities.

In addition, as a preliminary analysis, the homoscedasticity assumption was assessed using Levene's test. The Levene's test indicates the existence or inexistence of a significance violation of equality of variances. In other words, this test checks whether k samples present this homogeneity of variances (i.e. homoscedasticity).

#### 2.8.2 Primary and supplementary analysis (Friedman test and post-hoc test)

In the area artificial intelligence studies, particularly in modeling, multiple comparisons with a control method is one of the most commonly used statistical procedures [60]. In fact, the control method can be the most interesting algorithm for the researchers, what is commonly known as a novel proposed algorithm. When dealing with multiple comparisons tests, in statistical terminology, a block is composed of at least three results or subjects, every one corresponding to the performance evaluation of an algorithm or method based on a data set. Friedman test is a multiple comparisons non-parametric test equivalent to the repeated measures analysis of the variances, which can be used in this context as a primary analysis [54]. On the basis of the null-hypothesis, Friedman test states that all the algorithms have the equivalent performance, so a rejection of null-hypothesis implies the existence of significant differences among the performance of at least two algorithms.

Once Friedman's test rejects the null hypothesis, evaluating process can then be proceeded with a post-hoc test (i.e. a set/family of pairwise multiple comparisons tests) [61]. Indeed, the post-hoc test can be performed as a supplementary analysis in order to find out the significant differences between the performance of the control and novel proposed algorithm against the rest of the used algorithms. The post-hoc test statistic for comparing the i-th and j-th algorithm is given by Equation 54 [56].
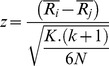
(54)where 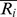 stands for the mean ranks calculated through the Friedman test for the i-th algorithm, k stands for the number of classifiers to be compared and N stands for the number of data sets used in the comparison. Here, the z-statistic value is used to determine the corresponding probability (p-value) from the table of standard normal distribution, which is then compared with an appropriate α.

In performing the post-hoc tests, controlling global Type I error, i.e. the so-called family-wise error rate (FWER) is a key feature [61]. More precisely, the FWER is the probability of rejecting at least one true null hypothesis among a family of pairwise multiple comparisons tests. The classic Bonferroni procedure is an appealing approach for the control of the FWER (i.e. to maintain FWER≤α) due to its applicability in various situations [62]. In practice, Bonferroni procedure controls FWER by adjusting p-values obtained from the post hoc test. On the other hand, the original Bonferroni procedure is generally considered as a conservative procedure. In order to overcome this issue, some modifications of the original Bonferroni procedure have been presented in the literature that are much more powerful than the conventional Bonferroni procedure [63], [64]. In this study, the focous was on Holm's sequentially rejective step down procedure that is a modified Bonferroni-based procedure for determining the adjusted p-value.

## Results

To evaluate the performance of the new proposed model, called the Hybrid Model, in this study, the seven standard data sets (i.e. five binary-class and two multi-class) were used. By using these data sets and based on some of the most commonly used evaluation criteria, the classification performance of the proposed model was compared to thirteen well-known classification methods. The contents are Adaptive Network Fuzzy Interface System (ANFIS), Radial Basis Function (RBF), k-NN, DWk-NN, Partial Distance k-NN (PDk-NN), MLP, Naive Bayes (NB), BPNN, Iterative Dichotomiser 3 (ID3), Bagging ID3 and Generalized Linear Models (GLM) with four different distributions.

### 3.1 Binary-class results

Based on the five binary-class data sets, the classification was performed using the proposed model and all the other thirteen methods mentioned previously. The performance of the methods was subsequently evaluated in terms of the overall classification accuracy, sensitivity, specificity, precision, F-measure and MCC on the basis of the confusion matrix results. The experimental results, as presented in Table 15, indicate that the proposed hybrid model has significantly outperformed all the other methods in terms of all the considered evaluation criteria for all the five binary-class data sets.

**Table 15:** The assessment results of the proposed model in comparison with the all other thirteen methods based on the five binary-class data sets by applying the six commonly used performance evaluation criteria.

Data sets		performances evaluation criteria					
	Name of Classifier	Accuracy	Sensitivity	Specificity	Precision	F-score	MCC
**Cleveland dataset (Binary-class)**	ANFIS	0.774	0.865	0.671	0.753	0.803	0.551
	NB	0.751	0.774	0.725	0.761	0.765	0.501
	BPNN	0.805	0.837	0.769	0.799	0.818	0.609
	GLM binomial	0.821	0.821	0.823	0.839	0.827	0.642
	GLM inv. gaussian	0.814	0.901	0.666	0.77	0.845	0.639
	GLM normal	0.843	0.903	0.773	0.824	0.86	0.687
	GLM poisson	0.848	0892	0.751	0.817	0.868	0.698
	ID3	0.744	0.761	0.727	0.769	0.761	0.491
	Bagging-ID3	0.804	0.844	0.758	0.805	0.822	0.606
	k-NN	0.801	0.825	0.775	0.812	0.815	0.602
	DWk-NN	0.811	0.834	0.784	0.817	0.823	0.621
	PDk-NN	0.802	0.837	0.76	0.799	0.816	0.599
	RBF	0.757	0.853	0.645	0.734	0.787	0.517
	Proposed Model	0.856	0.906	0.801	0.841	0.872	0.714
**Hungarian dataset**	ANFIS	0.806	0.894	0.651	0.818	0.853	0.572
	NB	0.779	0.785	0.769	0.856	0.817	0.542
	BPNN	0.787	0.883	0.618	0.813	0.840	0.517
	GLM binomial	0.815	0.862	0.733	0.846	0.853	0.601
	GLM inv. gaussian	0.801	0.927	0.585	0.794	0.854	0.564
	GLM normal	0.825	0.903	0.685	0.831	0.868	0.610
	GLM poisson	0.820	0.911	0.658	0.827	0.866	0.600
	ID3	0.768	0.824	0.670	0.819	0.819	0.497
	Bagging-ID3	0.803	0.869	0.695	0.827	0.846	0.577
	k-NN	0.778	0.874	0.611	0.801	0.834	0.511
	DWk-NN	0.783	0.867	0.633	0.808	0.835	0.521
	PDk-NN	0.784	0.875	0.633	0.803	0.836	0.530
	RBF	0.748	0.826	0.607	0.806	0.807	0.438
	Proposed Model	0.842	0.935	0.678	0.839	0.883	0.652
**WBC dataset**	ANFIS	0.908	0.965	0.799	0.901	0.931	0.795
	NB	0.938	0.956	0.903	0.948	0.952	0.863
	BPNN	0.931	0.923	0.946	0.971	0.946	0.854
	GLM binomial	0.972	0.961	0.973	0.976	0.968	0.941
	GLM inv. gaussian	0.917	0.979	0.783	0.892	0.933	0.821
	GLM normal	0.961	0.978	0.927	0.961	0.971	0.915
	GLM poisson	0.949	0.979	0.881	0.938	0.958	0.889
	ID3	0.949	0.959	0.931	0.962	0.961	0.889
	Bagging-ID3	0.968	0.971	0.965	0.981	0.976	0.932
	k-NN	0.960	0.977	0.953	0.974	0.975	0.932
	DWk-NN	0.967	0.976	0.950	0.973	0.974	0.927
	PDk-NN	0.968	0.974	0.957	0.976	0.975	0.931
	RBF	0.921	0.971	0.831	0.914	0.942	0.825
	Proposed Model	0.981	0.982	0.978	0.988	0.985	0.957
**WDBC dataset**	ANFIS	0.916	0.955	0.852	0.915	0.934	0.820
	NB	0.954	0.972	0.923	0.955	0.964	0.902
	BPNN	0.907	0.932	0.866	0.927	0.920	0.801
	GLM binomial	0.947	0.949	0.944	0.965	0.957	0.889
	GLM inv. gaussian	0.889	0.996	0.711	0.851	0.917	0.772
	GLM normal	0.946	0.987	0.878	0.931	0.958	0.885
	GLM poisson	0.931	0.994	0.825	0.905	0.947	0.855
	ID3	0.928	0.939	0.909	0.946	0.942	0.847
	Bagging-ID3	0.944	0.962	0.914	0.951	0.956	0.881
	k-NN	0.968	0.989	0.932	0.961	0.975	0.932
	DWk-NN	0.966	0.988	0.929	0.958	0.973	0.927
	PDk-NN	0.963	0.987	0.922	0.956	0.971	0.921
	RBF	0.954	0.983	0.907	0.946	0.964	0.903
	Proposed Model	0.983	0.998	0.959	0.976	0.987	0.965
**Pima dataset**	ANFIS	0.696	0.797	0.511	0.751	0.773	0.319
	NB	0.747	0.896	0.471	0.759	0.822	0.415
	BPNN	0.741	0.890	0.463	0.761	0.821	0.383
	GLM binomial	0.761	0.861	0.581	0.790	0.824	0.463
	GLM inv. gaussian	0.769	0.926	0.479	0.767	0.839	0.471
	GLM normal	0.769	0.893	0.539	0.783	0.835	0.471
	GLM poisson	0.765	0.896	0.521	0.777	0.832	0.459
	ID3	0.702	0.775	0.571	0.768	0.772	0.347
	Bagging-ID3	0.747	0.829	0.593	0.793	0.811	0.432
	k-NN	0.739	0.848	0.535	0.775	0.809	0.405
	DWk-NN	0.737	0.846	0.536	0.773	0.808	0.403
	PDk-NN	0.728	0.838	0.523	0.767	0.801	0.381
	RBF	0.759	0.889	0.521	0.774	0.828	0.449
	Proposed Model	0.774	0.936	0.611	0.797	0.861	0.481

### 3.2 Analysis of the conditions for a safe use of parametric tests on the binary-class results

The normality test of Shapiro-Wilk was performed on the obtained results by applying six commonly used classification evaluation criteria for assessing the performance of the fourteen methods, based on the five binary-class dataset at a significance level of α = 0.05. The results of the Shapiro-Wilk test show that the normality conditions are not fulfilled in some cases, they are not presented here to avoid reader confusion with so many results.

In order to verify the homoscedasticity hypothesis, Levene's test was performed on the results obtaining from the six classification evaluation criteria based on the five binary-class dataset at a significance level of α = 0.05. In effect, Levene's test is used for checking whether the fourteen used methods exhibit (or not) the homogeneity of variances. The results (p-values) are shown in Table 16, where the symbol “*” implies that homoscedasticity condition was not satisfied for a certain data set and a certain performance evaluation criterion.

**Table 16:** The homoscedasticity test results of Levene on the results were obtained from, the six performance evaluation criteria based on five binary-class dataset.

	performances evaluation criteria					
Name of dataset	Accuracy	Sensitivity	Specificity	Precision	F-score	MCC
**Cleveland**	0.00 *	0.00 *	0.00 *	0.00 *	0.00 *	0.01 *
**Hungarian**	0.03 *	0.00 *	0.00 *	0.20	0.00 *	0.01 *
**WBC**	0.02 *	0.00 *	0.00 *	0.01*	0.00 *	0.00 *
**WDBC**	0.01 *	0.00 *	0.00 *	0.02 *	0.00 *	0.00 *
**Pima**	0.03 *	0.00 *	0.00 *	0.10	0.00 *	0.01 *

The symbol “*” implies that homoscedasticity condition was not satisfied (the Levene's test was statistically significant (P-V<0.05)).

Based on the results of the normality and homoscedasticity tests, it can be concluded that the necessary conditions for the utilization of the parametric tests are not fulfilled in some cases. Thus, for testing the null-hypothesis that all the methods have similar performance applying non-parametric tests is appropriate.

### 3.3 Friedman and post-hoc tests' results for multiple comparisons on the binary-class results

The Friedman test was carried out on the results (i.e., the results of the classification performance of the proposed hybrid model and the all the other thirteen methods) were obtained from the six classification evaluation criteria based on the five binary-class dataset at a significance level of α = 0.05. The results, including test statistics and p-values, are presented in Table 17, where the symbol “*” implies that the null hypothesis was rejected for a certain performance evaluation criterion. As illustrated in Table 17, there are statistically significant differences between the algorithms' performance in terms of all the six classification evaluation criteria. Accordingly, in order to illustrate the significant differences between the performances of the proposed model against the rest of the used algorithms more concretely, the post hoc test was performed. Subsequently, the p-values resulting from the post hoc test were adjusted using Bonferroni–Holm's procedure. The post hoc tests results, including Z-Score, unadjusted p-value, coefficient adjustment of Holm and adjusted p-value, for all the six performance evaluation criteria are presented in Tables 18. It is apparent from the table that there are significant differences between the performances of the proposed model against the rest of considered algorithms in terms of each of the six classification evaluation criteria. In other words, the proposed model has significantly outperformed all the other algorithms in terms of all the considered evaluation criteria.

**Table 17:** The multiple comparison test results of Friedman on the results were obtained from, the six performance evaluation criteria based on the five binary-class data sets.

	performances evaluation criteria					
	Accuracy	Sensitivity	Specificity	Precision	F-score	MCC
**Friedman test statistics**	32.872	46.399	43.175	32.904	29.902	34.354
**P-Value**	0.001781 *	0.000012 *	0.000042 *	0.001761 *	0.001761*	0.001063 *

The symbol “*” implies that the Friedman's test was statistically significant (P-V<0.05).

**Table 18:** The pairwise multiple comparisons (post-hoc) test results of Holm (the proposed hybrid model (control algorithm) vs. the rest algorithms) on the results were obtained from, the six performance evaluation criteria based on the five binary-class data sets.

Evaluation criteria	Name of Classifier	Z-Score	P-Value	Coefficient adjustment of Holm	Adjusted P-Value
**Accuracy**	**Proposed Hybrid Model VS.**				
	ANFIS	4.5356	.000006	13	.000076*
	RBF	4.1576	.000032	12	.000388*
	ID3	3.9686	.000073	11	.000798*
	NB	3.7796	.000157	10	.001573*
	GLM inv. gaussian	3.5907	.000330	9	.002970*
	BPNN	3.4017	.000670	8	.005359*
	k-NN	3.4017	.000670	7	.004689*
	PDk-NN	3.1749	.001499	6	.008993*
	DWk-NN	3.0237	.002497	5	.012484*
	GLM binomial	2.8347	.004587	4	.018346*
	GLM poisson	2.6458	.008150	3	.024449*
	GLM normal	2.4568	.014018	2	.028036*
	Bagging-ID3	2.2678	.023341	1	.023341*
**Sensitivity**	**Proposed Hybrid Model VS.**				
	NB	4.5356	.000006	13	.000076*
	GLM binomial	4.1576	.000032	12	.000388*
	ID3	3.9686	.000073	11	.000798*
	ANFIS	3.7796	.000157	10	.001573*
	RBF	3.4017	.000670	9	.006028*
	BPNN	3.2127	.001315	8	.010519*
	DWk-NN	3.0993	.001940	7	.013578*
	PDk-NN	3.0237	.002497	6	.014981*
	k-NN	2.8347	.004587	5	.022933*
	GLM normal	2.6458	.008150	4	.032598*
	GLM poisson	2.5702	.010164	3	.030491*
	Bagging-ID3	2.4568	.014018	2	.028036*
	GLM inv. gaussian	2.2678	.023341	1	.023341*
**Specificity**	**Proposed Hybrid Model VS.**				
	GLM inv. gaussian	4.6868	.000003	13	.000037*
	RBF	3.9686	.000073	12	.000870*
	ANFIS	3.8552	.000116	11	.001275*
	GLM poisson	3.3639	.000769	10	.007686*
	BPNN	3.4017	.000670	9	.006028*
	NB	3.0993	.001940	8	.015517*
	ID3	3.0237	.002497	7	.017478*
	PDk-NN	2.9103	.003611	6	.021664*
	GLM normal	2.6458	.008150	5	.040748*
	k-NN	2.3434	.019109	4	.076436*
	DWk-NN	2.4568	.014018	3	.042054*
	Bagging-ID3	2.2678	.023341	2	.046683*
	GLM binomial	2.1544	.031209	1	.031209*
**Precision**	**Proposed Hybrid Model VS.**				
	ANFIS	4.2332	.000023	13	.000302*
	GLM inv. gaussian	4.1198	.000038	12	.000458*
	RBF	3.5151	.000440	11	.004838*
	PDk-NN	2.8725	.004072	10	.040721*
	DWk-NN	2.6458	.008150	9	.073346*
	NB	2.6080	.009107	8	.072857*
	BPNN	2.5702	.010164	7	.071146*
	GLM poisson	2.5702	.010164	6	.060983*
	GLM normal	2.4946	.012610	5	.063049*
	ID3	2.4568	.014018	4	.056072*
	k-NN	2.3434	.019109	3	.057327*
	GLM binomial	2.2678	.023341	2	.046683*
	Bagging-ID3	2.2300	.025748	1	.025748*
**F-Score**	**Proposed Hybrid Model VS.**				
	ID3	4.5356	.000006	13	.000076*
	ANFIS	4.1576	.000032	12	.000388*
	RBF	3.7796	.000157	11	.001731*
	BPNN	3.2883	.001008	10	.010080*
	NB	3.2505	.001152	9	.010368*
	GLM inv. gaussian	3.0237	.002497	8	.019975*
	k-NN	2.9103	.003611	7	.025274*
	PDk-NN	2.8347	.004587	6	.027520*
	DWk-NN	2.7213	.006502	5	.032512*
	Bagging-ID3	2.6458	.008150	4	.032598*
	GLM poisson	2.4568	.014018	3	.042054*
	GLM binomial	2.3434	.019109	2	.038218*
	GLM normal	2.2678	.023341	1	.023341*
**MCC**	**Proposed Hybrid Model VS.**				
	ID3	4.1198	.000038	13	.000496*
	ANFIS	3.9308	.000085	12	.001019*
	BPNN	3.6285	.000285	11	.003138*
	NB	3.4017	.000670	10	.006698*
	RBF	3.3261	.000881	9	.007927*
	GLM inv. gaussian	3.0993	.001940	8	.015517*
	PDk-NN	3.0237	.002497	7	.017478*
	k-NN	2.9103	.003611	6	.021664*
	DWk-NN	2.8347	.004587	5	.022933*
	GLM poisson	2.6458	.008150	4	.032598*
	GLM normal	2.4568	.014018	3	.042054*
	Bagging-ID3	2.3812	.017256	2	.034513*
	GLM binomial	2.3056	.021133	1	.021133*

The symbol “*” implies that pairwise multiple comparisons (post-hoc) test was statistically significant (P-V<0.05).

### 3.4 Multi-class results

On the basis of two multi-class dataset, the classification was carried out by the same way described for binary-class data sets. The performance evaluation criteria was subsequently determined for each classifier based on the confusion matrix results. The results are shown in Table 19 in terms of overall classification accuracy, P-micro, P-macro, R-micro, R-macro, F-micro, F-macro, MCC and 1-CEN. The experimental results in Table 19 have demonstrated that the proposed hybrid model has significantly outperformed all the other algorithms in terms of all considered evaluation criteria for the two multi-class data sets.

**Table 19:** The assessment results of the proposed model in comparison with the all other thirteen methods based on the two multi-class data sets by applying the nine commonly used performance evaluation criteria.

Data sets		performances evaluation criteria								
	Name of Classifier	Accuracy	P-micro	P-macro	R-micro	R-macro	F-micro	F-macro	MCC	1-CEN
**Cleveland dataset (Multi-class)**	**ANFIS**	0.546	0.365	0.352	0.378	0.351	0.371	0.327	0.297	0.579
	NB	0.525	0.331	0.312	0.323	0.301	0.327	0.290	0.264	0.578
	BPNN	0.588	0.364	0.350	0.329	0.314	0.346	0.275	0.408	0.646
	GLM binomial	0.613	0.371	0.357	0.357	0.344	0.364	0.291	0.360	0.711
	GLM inv. gaussian	0.584	0.353	0.347	0.349	0.332	0.351	0.287	0.291	0.697
	GLM normal	0.613	0.358	0.343	0.361	0.348	0.359	0.288	0.364	0.713
	GLM poisson	0.594	0.351	0.332	0.342	0.329	0.346	0.284	0.328	0.696
	ID3	0.505	0.321	0.308	0.325	0.304	0.323	0.290	0.242	0.541
	Bagging-ID3	0.593	0.378	0.361	0.359	0.342	0.368	0.332	0.279	0.601
	k-NN	0.559	0.321	0.294	0.298	0.285	0.309	0.268	0.294	0.612
	DWk-NN	0.561	0.319	0.311	0.312	0.299	0.315	0.286	0.296	0.597
	PDk-NN	0.569	0.325	0.318	0.322	0.304	0.323	0.292	0.289	0.598
	RBF	0.531	0.285	0.261	0.218	0.209	0.247	0.157	0.050	0.721
	Proposed Model	0.621	0.423	0.507	0.431	0.418	0.427	0.436	0.497	0.804
**ACSEKI dataset**	ANFIS	0.501	0.483	0.278	0.512	0.499	0.497	0.207	0.252	0.839
	NB	0.507	0.502	0.481	0.451	0.448	0.475	0.357	0.348	0.669
	BPNN	0.776	0.651	0.332	0.534	0.250	0.587	0.183	0.001	0.770
	GLM binomial	0.563	0.493	0.487	0.341	0.325	0.403	0.319	0.215	0.659
	GLM inv. gaussian	0.621	0.495	0.489	0.412	0.401	0.450	0.392	0.356	0.674
	GLM normal	0.547	0.491	0.484	0.324	0.319	0.390	0.303	0.206	0.655
	GLM poisson	0.582	0.523	0.516	0.361	0.353	0.427	0.344	0.267	0.665
	ID3	0.915	0.781	0.765	0.768	0.763	0.774	0.763	0.856	0.892
	Bagging-ID3	0.937	0.793	0.773	0.752	0.745	0.772	0.741	0.894	0.926
	k-NN	0.777	0.656	0.648	0.591	0.580	0.622	0.584	0.604	0.751
	DWk-NN	0.784	0.642	0.637	0.589	0.585	0.614	0.590	0.615	0.756
	PDk-NN	0.763	0.607	0.598	0.576	0.569	0.591	0.574	0.579	0.729
	RBF	0.621	0.421	0.407	0.352	0.345	0.383	0.276	0.184	0.777
	Proposed Model	0.952	0.868	0.784	0.812	0.792	0.839	0.774	0.915	0.931

### 3.5 Analysis of the conditions for a safe use of parametric tests on the multi-class results

The normality test of Shapiro-Wilk was carried out on the obtained results by applying the nine evaluation criteria for assessing the performance of the fourteen methods, based on the two multi-class dataset at a significance level of α = 0.05. The results of the Shapiro-Wilk test show that the normality conditions are not fulfilled in some cases, they are not presented here to avoid reader confusion with so many results.

In order to verify the homoscedasticity hypothesis, Levene's test was carried out on the results obtaining from the nine classification evaluation criteria based on the two multi-class data sets at a significance level of α = 0.05. The results (p-value) were presented in Table 20, where the symbol “*” implies that homoscedasticity condition was not satisfied for a certain data set and a certain performance evaluation criterion. Based on the results of the normality and homoscedasticity tests, it can be concluded that the necessary conditions for the utilization of parametric tests are not fulfilled in some cases. Thus, for testing the null-hypothesis that all the methods have similar performance, applying non-parametric tests is appropriate.

**Table 20:** The homoscedasticity test results of Levene on the results were obtained from, the six performance evaluation criteria based on five binary-class dataset.

	performances evaluation criteria								
Name of Classifier	Accuracy	P-micro	P-macro	R-micro	R-macro	F-micro	F-macro	MCC	1-CEN
**Cleveland**	0,00*	0.04*	0.02*	0.01*	0.00*	.03*	0.00*	0.17	0.13
**ACSEKI**	0,00*	0.01*	0.11*	0.00*	0.00*	.06	0.00*	0.02*	0.04*

The symbol “*” implies that homoscedasticity condition was not satisfied (the Levene's test was statistically significant (P-V<0.05)).

### 3.6 Friedman and post-hoc tests' results for multiple comparisons on the multi-class results

The Friedman test was performed on the results obtained from the nine classification evaluation criteria based on the two multi -class dataset at a significance level of α = 0.05. The experimental results, including test statistics and p-values, are shown in Table 21, where the symbol “*” implies that the null hypothesis was rejected for a certain performance evaluation criterion.

**Table 21:** The multiple comparisons test results of Friedman on the results were obtained from, the nine performance evaluation criteria based on two multi-class data sets.

	performances evaluation criteria								
Name of Classifier	Accuracy	P-micro	P-macro	R-micro	R-macro	F-micro	F-macro	MCC	1-CEN
**Friedman test statistics**	23.614	24.647	22.771	24.068	24.056	23.492	29.24	30.375	32.571
**P-Value**	.035*	.026*	.045*	.031*	.031*	.036*	.006*	.004*	.002*

The symbol “*” implies that the Friedman's test was statistically significant (P-V<0.05).

As it is shown in Table 21, there are statistically significant differences between the algorithms performance on the basis of all the nine classification evaluation criteria. Accordingly, in order to more concretely depict the significant differences between the performances of the proposed model and the rest of used algorithms, a post hoc test was performed. Subsequently, the p-values resulting from the post hoc test were adjusted using Bonferroni–Holm's procedure. The results of the post hoc tests, including Z-Score, unadjusted p-value, coefficient adjustment of Holm and adjusted p-value for all the nine performance evaluation criteria are presented in Table 22. Some of the highlights of the table are outlined as follows. As expected, it is found that the overall proposed model significantly outperformed the other used algorithms.

**Table 22:** The pairwise multiple comparisons (post-hoc) test results of Holm (the proposed hybrid model (control algorithm) vs. the rest algorithms) on the results were obtained from, the nine performance evaluation criteria based on the two multi-class data sets.

Evaluation criteria	Name of Classifier	Z-Score	P-Value	Coefficient adjustment of Holm	Adjusted P-Value
**Accuracy**	**Hybrid Model VS.**				
	NB	4.5356	.000006	13	.000075*
	ANFIS	4.5356	.000006	12	.000069*
	RBF	4.0329	.000055	11	.000606*
	GLM normal	3.7154	.000203	10	.002029*
	GLM binomial	3.4659	.000528	9	.004756*
	GLM poisson	3.4017	.000670	8	.005357*
	GLM inv. gaussian	3.2770	.001049	7	.007344*
	PDk-NN	3.1371	.001706	6	.010238*
	k-NN	3.0237	.002497	5	.012485*
	ID3	2.9481	.003197	4	.012789*
	DWk-NN	2.8990	.003744	3	.011231*
	BPNN	1.7651	.077547	2	.155094
	Bagging-ID3	1.1339	.256837	1	.256837
**P-micro**	**Hybrid Model VS.**				
	RBF	4.1576	.000032	13	.000418*
	NB	3.9686	.000072	12	.000868*
	ANFIS	3.7796	.000157	11	.001728*
	GLM normal	3.4017	.000670	10	.006697*
	GLM binomial	3.2127	.001315	9	.011834*
	GLM poisson	3.0993	.001940	8	.015518*
	k-NN	3.0237	.002497	7	.017479*
	GLM inv. gaussian	2.9481	.003197	6	.019184*
	PDk-NN	2.8725	.004072	5	.020362*
	DWk-NN	2.7705	.005597	4	.022388*
	ID3	1.8256	.067911	3	.203732
	BPNN	1.5119	.130559	2	.261119
	Bagging-ID3	.3780	.705431	1	.705431
**P-macro**	**Hybrid Model VS.**				
	RBF	4.4108	.000010	13	.000134*
	NB	3.6549	.000257	12	.003087*
	ANFIS	3.6549	.000257	11	.002830*
	BPNN	3.5264	.000421	10	.004213*
	GLM normal	3.4017	.000670	9	.006027*
	GLM poisson	3.3261	.000881	8	.007046*
	GLM binomial	3.1371	.001706	7	.011944*
	k-NN	3.0237	.002497	6	.014982*
	DWk-NN	2.9103	.003611	5	.018054*
	PDk-NN	2.8347	.004587	4	.018347*
	GLM inv. gaussian	2.6458	.008169	3	.024507*
	ID3	1.8898	.058785	2	.117569
	Bagging-ID3	.3780	.001706	1	.001706
**R-micro**	**Hybrid Model VS.**				
	GLM normal	4.5356	.000006	13	.000075*
	RBF	4.4108	.000010	12	.000124*
	GLM binomial	3.4017	.000670	11	.007366*
	GLM poisson	3.1484	.001642	10	.016417*
	NB	3.0237	.002497	9	.022473*
	GLM inv. gaussian	3.0237	.002497	8	.019976*
	k-NN	3.0237	.002497	7	.017479*
	ID3	2.9481	.003197	6	.019184*
	DWk-NN	2.8347	.004587	5	.022934*
	PDk-NN	2.7969	.005160	4	.020638*
	ANFIS	2.7213	.006503	3	.019508*
	BPNN	1.6366	.101714	2	.203428
	Bagging-ID3	1.2586	.208175	1	.208175
**R-macro**	**Hybrid Model VS.**				
	RBF	4.5356	.000006	13	.000075*
	BPNN	4.0442	.000053	12	.000630*
	NB	3.4017	.000670	11	.007366*
	k-NN	3.4017	.000670	10	.006697*
	DWk-NN	2.9103	.003611	9	.032497*
	GLM poisson	2.7591	.005796	8	.046369*
	GLM normal	2.7213	.006503	7	.045518*
	PDk-NN	2.7213	.006503	6	.039015*
	GLM binomial	2.6458	.008150	5	.040749*
	GLM inv. gaussian	2.6458	.008150	4	.032599*
	ID3	1.5875	.112399	3	.337198*
	ANFIS	1.1339	.256837	2	.513673
	Bagging-ID3	1.0583	.289919	1	.289919
**F-micro**	**Hybrid Model VS.**				
	RBF	4.9135	.000001	13	.000012*
	GLM normal	4.5356	.000006	12	.000070*
	GLM poisson	4.1576	.000032	11	.000356*
	GLM binomial	3.9686	.000073	10	.000725*
	NB	3.7796	.000157	9	.001416*
	GLM inv. gaussian	3.4017	.000670	8	.005359*
	PDk-NN	3.0237	.002497	7	.017478*
	DWk-NN	2.9103	.003611	6	.021664*
	k-NN	2.8347	.004587	5	.022933*
	ANFIS	2.7213	.006502	4	.026009*
	BPNN	2.6458	.008150	3	.024449*
	ID3	1.5761	.115003	2	.230005
	Bagging-ID3	.6312	.527910	1	.527910
**F-macro**	**Hybrid Model VS.**				
	RBF	4.6868	.000003	13	.000037*
	BPNN	4.5356	.000006	12	.000070*
	GLM normal	4.1576	.000032	11	.000356*
	GLM poisson	3.9686	.000073	10	.000725*
	ANFIS	3.7796	.000157	9	.001416*
	GLM binomial	3.4017	.000670	8	.005359*
	k-NN	3.2127	.001315	7	.009205*
	GLM inv. gaussian	3.0993	.001940	6	.011638*
	NB	3.0237	.002497	5	.012484*
	DWk-NN	2.8347	.004587	4	.018346*
	PDk-NN	2.7213	.006502	3	.019507*
	ID3	2.6458	.008150	2	.016299*
	Bagging-ID3	.5292	.596667	1	.596667
**MCC**	**Hybrid Model VS.**				
	RBF	4.8379	.000001	13	.000018*
	NB	4.5356	.000006	12	.000070*
	GLM normal	4.3466	.000014	11	.000154*
	GLM poisson	4.1576	.000032	10	.000324*
	GLM binomial	3.8930	.000099	9	.000893*
	GLM inv. gaussian	3.7796	.000157	8	.001259*
	PDk-NN	3.6663	.000246	7	.001724*
	BPNN	3.5907	.000330	6	.001980*
	ANFIS	3.4017	.000670	5	.003349*
	k-NN	3.3261	.000881	4	.003523*
	DWk-NN	3.0237	.002497	3	.007491*
	ID3	2.6458	.008150	2	.016299*
	Bagging-ID3	2.0788	.037636	1	.037636*
**1-CEN**	**Hybrid Model VS.**				
	NB	4.5356	.000006	13	.000076*
	PDk-NN	4.1576	.000032	12	.000388*
	DWk-NN	3.9686	.000073	11	.000798*
	GLM poisson	3.7796	.000157	10	.001573*
	k-NN	3.6663	.000246	9	.002217*
	GLM normal	3.5907	.000330	8	.002640*
	GLM binomial	3.4017	.000670	7	.004689*
	GLM inv. gaussian	3.2505	.001152	6	.006912*
	ANFIS	3.0237	.002497	5	.012484*
	BPNN	2.8347	.004587	4	.018346*
	ID3	2.6458	.008150	3	.024449*
	Bagging-ID3	2.2678	.023341	2	.046683*
	RBF	2.0410	.041251	1	.041251*

The symbol “*” implies that pairwise multiple comparisons (post-hoc) test was statistically significant (P-V<0.05).

More specifically, there are significant differences between the performances of the proposed model and the rest of the considered algorithms (i.e., the proposed model significantly outperformed the other algorithms) in terms of the MCC and 1-CEN criteria. Moreover, the proposed model achieved higher classification accuracy than 11 out of the 13 considered algorithms (i.e., except the BPNN and Bagging-ID3). Furthermore, the experimental results represented a meaningful improvement of the proposed model over all the used algorithms except the ID3, BPNN and Bagging-ID3 in terms of P-micro. Besides, it is apparent that the proposed model has a higher performance than all the other used algorithms except the ID3 and Bagging-ID3 in terms of P-macro. In addition, the results revealed the superiority of the proposed model over all the other considered methods except the BPNN and in terms of R-micro. Also, it can be concluded that the performance of the proposed model surpasses all the other considered methods except ANFIS and Bagging-ID3 on the basis of R-macro. Finally, based on F-micro and F-macro criteria, the proposed model demonstrated superior performance for 11 out of the 13 (i.e., except the ID3 and Bagging-ID3) and 12 out of the 13 considered algorithms respectively (i.e., except the Bagging-ID3).

### 3.7 Comparison with the other state-of-the-art models

In this section, the obtained results of the proposed hybrid model are compared to the obtained results of the state-of-the-art classifiers (i.e., the single, hybrid or ensemble SVM and random forest-based models) in the recent literature in terms of classification accuracy for the data sets under consideration. The results of this comprehensive comparative survey are reported in Table 23. It is apparent from the table that the proposed model shows very promising performance. More specifically, as shown in Table 23, the proposed model demonstrated superior classification accuracy for 13 out of the 14 algorithms in multi-class Cleveland dataset.

**Table 23:** Classification accuracies obtained with the proposed hybrid model and the other state-of-the-art classifiers from the recent literature for the data sets under consideration.

Data sets	Kind of Hybrid	Author	Name of Classifier	Year	Accuracy
**Cleveland dataset (Multi-class)**	**RF based Hybrid**	Zhang et al.	RF	2008	55.62
		Zhang et al.	RF- AdaBoost	2008	56.20
		Ghaemi et al.	FW-FOA	2014	58.14
					
	**SVM Based Hybrid**	Madhu et al.	SVM-ZDISC	2014	57.90
		Madhu et al.	SVM-Bayesian	2014	56.08
		Madhu et al.	SVM-Fayyad-Irani	2014	57.74
		Madhu et al.	SVM-CACC	2014	56.70
		Forghani et al.	SVM-Fuzzy	2014	63.00
	**Other Hybrid**	Zhang et al.	AdaBoost	2008	54.45
		Zhang et al.	MultiBoost	2008	55.52
		Madhu et al.	C4.5-ZDISC	2014	57.09
		Madhu et al.	C4.5-Bayesian	2014	52.50
		Madhu et al.	C4.5-Fayyad-Irani	2014	57.97
		Madhu et al.	C4.5-CACC	2014	50.80
		**Proposed Model**	**Hybrid Model**	**2014**	**62.10**
**Cleveland dataset (Binary-class)**	**RF based Hybrid**	Tan et al.	SVM-GA	2009	84.07
		Ozcift	RF-CFS	2011	80.49
		Ballings et al.	RF	2013	82.12
		Ballings et al.	KIRF-RBF	2013	67.55
		Fernandez-Delgado et al.	RF	2014	80.40
	**SVM Based Hybrid**	Tan et al.	SVM-GA	2009	84.07
		Fernandez-Delgado et al.	SVM-DKP	2014	79.90
		Fernandez-Delgado et al.	SVM	2014	81.60
		Chen et al.	GRID-SVM	2014	83.44
		Chen et al.	PSO-SVM	2014	86.55
		Chen et al.	PTVPSO-SVM	2014	87.21
	**Other Hybrid**	Zhang et al.	LP-Adaboost	2011	77.04
		Zhang et al.	LP-WV	2011	83.22
		Zhang et al.	MCE-WV	2011	81.70
		Ahmad et al.	Improved GA-MLP	2013	85.50
		Ballings et al.	KF-RBF	2013	75.91
		**Proposed Model**	**Hybrid Model**	**2014**	**85.60**
**Hungarian dataset**					
	**Other Hybrid**	Rodriguez et al.	Resampling-AdaBoost	2008	80.96
		Rodriguez et al.	Reweighting -AdaBoost	2008	81.44
		Sarkar et al.	Naïve Bayes-GA	2012	73.30
		Sarkar et al.	C4.5-GA	2012	78.08
		Sarkar et al.	ANN-GA	2012	69.43
		**Proposed Model**	**Hybrid Model**	**2014**	**84.20**
**WDBC dataset**	**RF based Hybrid**	Yao et al.	RF_CFS	2011	96.26
		Yao et al.	RF-MARS	2011	96.29
		Ozcift	RF-Bayes Network	2012	96.22
		Ozcift	RF- Naive Bayes	2012	96.22
		Ozcift	RF- RBF	2012	96.22
		Ozcift	RF-kstar	2012	99.05
		Ozcift	RF- Logistics	2012	98.11
		Khan	RF-GA	2013	76.26
		Cadenas et al.	RF-Fuzzy	2013	95.20
		Cadenas et al.	RF-Fuzzy-fs	2013	95.25
	**SVM Based Hybrid**	Nandi et al.	SVM-SOM–RBF	2006	98.00
		Polat et al.	SVM- LS	2007	98.53
		Kumar et al.	SVM-DT	2010	87.08
		Chen et al.	SVM-RS	2011	96.87
		Chen et al.	PSO-SVM	2012	99.30
		Desir	RF-OC	2012	96.00
		Chaurasia et al.	SVM-CFS	2013	96.40
		Zheng et al.	K-means -SVM	2014	97.38
		Gorunescu et al.	SVM	2014	95.58
		Chen et al.	PSO-SVM	2014	98.01
		Chen et al.	PTVPSO-SVM	2014	98.44
		Chen et al.	GRID-SVM	2014	97.45
	**Other Hybrid**	Hassan et al.	Fuzzy-HMM	2010	98.16
		Chin et al.	Fuzzy tree-CB	2011	98.90
		Ballings et al.	KF-RBF	2013	94.19
		Gorunescu et al.	MLP-GA	2014	93.58
		**Proposed Model**	**Hybrid Model**	**2014**	**98.30**
**WBC dataset**	**RF based Hybrid**	Desir et al.	RF-One class	2012	96.00
		Seera et al.	RF-FuzzyMM-CART	2014	97.29
		Bonissone et al.	RF-Fuzzy	2010	97.30
		Bonissone et al.	RF	2010	97.07
	**SVM Based Hybrid**	Desir et al.	SVM-One class	2012	92.00
		Stoean et al.	SVM	2013	96.50
		Stoean et al.	SVM-FS	2013	97.07
		Fernandez-Delgado et al.	SVM-DKP	2014	96.10
		Fernandez-Delgado et al.	SVM	2014	97.10
		Gorunescu et al.	SVM	2014	96.92
		Chen et al.	PSO-SVM	2014	97.55
		Chen et al.	PTVPSO-SVM	2014	98.62
		Chen et al.	GRID-SVM	2014	96.62
	**Other Hybrid**	Bonissone et al.	Fuzzy tree-Bagging	2010	95.68
		Bonissone et al.	Fuzzy tree-Boosting	2010	94.51
		Polat et al.	Fuzzy-AIRS	2007	98.51
		Subashini et al.	SVM-CFS	2011	92.13
		Orkcu et al.	Binary Coded-GA	2011	94.00
		Luukka	similarity classifier	2011	97.49
		Luukka	similarity classifier-Fuzzy entropy	2011	97.18
		Gorunescu et al.	MLP-GA	2014	91.42
		Seera et al.	FuzzyMM-CART	2014	93.14
		**Proposed Model**	**Hybrid Model**	**2014**	**98.10**
**Pima dataset**	**RF based Hybrid**	Bonissone et al.	RF-Fuzzy	2010	76.53
		Bonissone et al.	RF	2010	75.26
		Desir et al.	RF-One class	2012	68.00
		Tripoliti et al.	RF	2012	77.30
		Cadenas et al.	RF-Fuzzy	2013	76.43
		Cadenas et al.	RF-Fuzzy-fs	2013	75.69
		Fernandez-Delgado et al.	RF	2014	74.60
		Seera et al.	RF-FuzzyMM-CART	2014	76.56
	**SVM Based Hybrid**	Polat et al.	GDA–LS-SVM	2008	82.05
		Tan et al.	SVM-GA	2009	78.26
		Desir et al.	SVM- One class	2012	34.00
		Chorowski et al.	SVM	2014	76.00
		Chorowski et al.	SVM-LS	2014	76.00
		Fernandez-Delgado et al.	SVM-DKP	2014	74.7
		Fernandez-Delgado et al.	SVM	2014	75.8
		Chen et al.	PSO-SVM	2014	77.58
		Chen et al.	PTVPSO-SVM	2014	78.14
		Chen et al.	GRID-SVM	2014	76.65
	**Other Hybrid**	Dogantekin et al.	LDA-ANFIS	2010	84.61
		Bonissone et al.	Fuzzy tree	2010	67.55
		Bonissone et al.	Fuzzy tree-Bagging	2010	73.63
		Bonissone et al.	Fuzzy tree-Boosting	2010	66.18
		Ozcift	RF-CFS	2011	74.47
		Luukka	similarity classifier	2011	75.29
		Luukka	similarity classifier-Fuzzy entropy	2011	75.97
		Chorowski et al.	ELM	2014	76.00
		Chorowski et al.	ML-ELM	2014	77.00
		Fernandez-Delgado et al.	SVM-DKP	2014	74.7
		Fernandez-Delgado et al.	SVM	2014	75.8
		Seera et al.	FuzzyMM-CART	2014	69.13
		**Proposed Model**	**Hybrid Model**	**2014**	**77.40**

Moreover, in binary-class Cleveland dataset, the accuracy of proposed model was competitive to 14 out of 16 the state-of-the-art classifiers. Furthermore, the proposed classification model in terms of accuracy is better than all six considered algorithms in Hungarian dataset. Besides, in WDBC and WBC datasets the classification accuracy of the proposed model surpasses 21 out of 26 and 20 out of 22 the state-of-the-art classifiers, respectively. Finally, in Pima Indian diabetes dataset our hybrid model obtains higher classification accuracies for 26 out of the 30 state-of-the-art classifiers.

## Conclusions

Classification models based on artificial intelligence have had a significant impact on the predictive decision making process in various sciences, including medicine. Numerous research have been carried out on these classification models. Nevertheless, research continues to achieve models with better efficiency. Combing different methods and algorithms to find more efficient hybrid models is an approach that has attracted a lot of attention. The basic and fundamental point in the structure of such models is the proper selection of their components to benefit from exclusive features of each method or algorithm in the hybrid model, as well as increasing the accuracy in their combination. Building such a combination and benefiting from the advantages of each method can eliminate the deficiencies of the participatory methods and create a hybrid model with the least deficiency.

The main goal of the present study was developing the accuracy of the classification models with take advantage of a synergy that was expected to emerge from the hybridization of the components of the proposed model. The proposed hybrid model was implemented based on combining some methods and algorithms of artificial intelligence in three main stages. First, selecting appropriate features and then creating optimized features' arrays from them using pattern recognition methods, and also unique features of GA in optimization. Second, performing the classification in parallel with the two methods of the modified K-NN and the BPNN, developed into DBPNN. Finally, the integration of the final decision of class allocation using the Fuzzy class membership.

It should be pointed out that in this study, the decision making process in class allocation was improved through an adjustment made in the last stage of the k-NN algorithm so that several sets of k-dimensional optimized arrays of features (i.e., generated by GA) were used instead of one set. In addition, a developed network called DBPNN was created by introducing a dynamic transfer function for BPNN. A problem that the transfer function of these networks such as logarithm and tangent Sigmoid functions suffer from is the limitation of their active domain. This means that those functions will have better performance in a limited range of their domains (active domain). Out of this range, the network has a rather stable output and its derivative is also very close to zero in these range. This would result in the slowing of the learning process and the reduction of the classifier's accuracy, both of which were resolved via this method.

The evaluation process of the proposed hybrid model was performed by repeated random sub-sampling cross validation and the method of three-way data splits on six data sets taken from the UCI machine-learning repository and another dataset in the real world called ACSEKI. Four instances of the data were related to heart disease, two instances were concerned to breast cancer and one instance was regarded to diabetes. In this evaluation process, by taking the seven data sets into account and based on some of the most commonly used evaluation criteria, the classification performance of the proposed model was compared with the thirteen of the most well-known classification methods.

The statistical analysis was performed using the non-parametric Friedman test and followed by post-hoc tests (i.e., the Dunn's pairwise multiple comparisons tests). The p-values resulted from the post hoc test were adjusted using Bonferroni–Holm's procedure. Interestingly, the experimental results indicated that the proposed hybrid model has significantly outperformed the all others thirteen considered classification methods, and effectively increased the classification accuracy as well. Furthermore, in a comprehensive comparative survey, the performance results of the proposed model were benchmarked against the best ones reported for the state-of-the-art classifiers (i.e., the single, hybrid or ensemble SVM-based and random forest-based classifiers) in the recent literature in terms of classification accuracy for the same data sets under consideration. This comparative survey has provided a concise summary of substantial results that reveal the efficiency of the proposed model is desirable, promising, and competitive to the state-of-the-art classification models available in the literature. It worth mentioning that the nature of hybrid models is associated with minor inevitable complexities and the proposed model in this study is not an exception. Nevertheless, given the proven capabilities and the effectiveness of this model in the classification duty in three different fields, that is in ACS, breast cancer and diabetes, there is hope that the proposed model could be used as a tool in the quick, timely, and accurate diagnosis of diseases in other medical fields as well as in non-medical ones.
